# Enzymatic activities and functional interdependencies of *Bacillus subtilis* lipoteichoic acid synthesis enzymes

**DOI:** 10.1111/j.1365-2958.2010.07472.x

**Published:** 2011-02

**Authors:** Mirka E Wörmann, Rebecca M Corrigan, Peter J Simpson, Steve J Matthews, Angelika Gründling

**Affiliations:** 1 Section of Microbiology, Imperial College LondonSouth Kensington Campus, London SW7 2AZ, UK.; 2 Division of Molecular Biosciences, Imperial College LondonSouth Kensington Campus, London SW7 2AZ, UK

## Abstract

Lipoteichoic acid (LTA) is an important cell wall polymer in Gram-positive bacteria. The enzyme responsible for polyglycerolphosphate LTA synthesis is LtaS, first described in *Staphylococcus aureus.* Four LtaS orthologues, LtaS_BS_, YfnI, YqgS and YvgJ, are present in *Bacillus subtilis.* Using an *in vitro* enzyme assay, we determined that all four proteins are Mn^2+^-dependent metal enzymes that use phosphatidylglycerol as a substrate. We show that LtaS_BS_, YfnI and YqgS can produce polymers, suggesting that these three proteins are bona-fide LTA synthases while YvgJ functions as an LTA primase, as indicated by the accumulation of a GroP-Glc_2_-DAG glycolipid. Western blot analysis of LTA produced by *ltaS_BS_*, *yfnI*, *yqgS* and *yvgJ* single, triple and the quadruple mutant, showed that LTA production was only abolished in the quadruple and the YvgJ-only expressing mutant. *B. subtilis* strains expressing YfnI in the absence of LtaS_BS_ produced LTA of retarded mobility, presumably caused by an increase in chain length as suggested by a structural analysis of purified LTA. Taken together, the presented results indicate that the mere presence or absence of LTA cannot account for cell division and sporulation defects observed in the absence of individual enzymes and revealed an unexpected enzymatic interdependency of LtaS-type proteins in *B. subtilis*.

## Introduction

Teichoic acids are important cell wall components in Gram-positive bacteria. Usually, two types of teichoic acids are present, wall teichoic acid (WTA), which is covalently linked to peptidoglycan and lipoteichoic acid (LTA), which is tethered to the membrane by a lipid anchor. WTA polymers are commonly made up of glycerol- or ribitolphosphate subunits, but tetroses, hexoses or complex sugar combinations have also been reported. LTA is usually less diverse and often consists of a glycerolphosphate chain retained by a glycolipid anchor in the bacterial membrane (Fischer, 1988; Fischer *et al*., 1990; Weidenmaier and Peschel, 2008). Both *S. aureus* and *B. subtilis* synthesize this type of LTA consisting of an unbranched 1,3-linked polyglycerolphosphate chain tethered to the bacterial membrane by a diglucosyl-diacylglycerol (Glc_2_-DAG) glycolipid (Duckworth *et al*., 1975; Fischer, 1988; 1994;). The glycerolphosphate subunits are esterified to a varying degree with d-alanine groups and glycosyl modifications are also present in many *Bacillus* sp. (Fischer and Rosel, 1980; Iwasaki *et al*., 1986; Fischer, 1988; 1994; Iwasaki *et al*., 1989). While the exact function of teichoic acids is not clear, they have distinct roles within the cell. The absence of WTA in *B. subtilis* leads to cell rounding (D'Elia *et al*., 2006), while the absence of LTA leads to the formation of long filaments that spiral along their long axes (Schirner *et al*., 2009). *S. aureus* cells lacking WTA show slight morphological alterations and are less virulent (Weidenmaier *et al*., 2004; 2005;), while the absence of LTA causes severe morphological defects and bacteria are only viable under certain growth conditions (Gründling and Schneewind, 2007a; Oku *et al*., 2009). However, a combined absence of WTA and LTA is lethal for both *S. aureus* and *B. subtilis* (Oku *et al*., 2009; Schirner *et al*., 2009), making enzymes involved in their synthesis promising new drug targets (Falconer and Brown, 2009).

The enzyme responsible for polyglycerolphosphate LTA backbone synthesis is LtaS, first described in *S. aureus* (Gründling and Schneewind, 2007a). LtaS is predicted to contain five N-terminal transmembrane helices followed by a large extracellular enzymatic domain (eLtaS). The full-length protein is cleaved during growth and the eLtaS domain is released into the culture supernatant as well as partially retained within the cell wall fraction (Lu *et al*., 2009). Pulse-chase experiments have provided strong experimental evidence that the glycerolphosphate subunits of the LTA backbone are derived from the membrane lipid phosphatidylglycerol (PG) (Emdur and Chiu, 1974; 1975; Koch *et al*., 1984). Using an *in vitro* enzyme assay system, we have recently shown that the purified *S. aureus* eLtaS domain is sufficient to cleave the head group of fluorescently labelled PG producing diacylglycerol (DAG) and presumably glycerolphosphate, providing further evidence that this lipid is the physiological substrate for LtaS and LTA synthesis (Karatsa-Dodgson *et al*., 2010).

Interestingly, *B. subtilis* contains four LtaS orthologues, namely LtaS_BS_ (YflE), YfnI, YqgS and YvgJ with more than 40% identity to *S. aureus* LtaS. All four proteins have the same predicted membrane topology and domain structure as *S. aureus* LtaS. In addition, all four proteins have an AXA motif following the transmembrane domain (Fig. S1), which is reminiscent of and has been predicted to be a signal peptidase cleavage site (Antelmann *et al*., 2001). Furthermore, in proteomic studies processed forms of LtaS_BS_ and YfnI were detected in the culture supernatant (Hirose *et al*., 2000; Tjalsma *et al*., 2004), showing that at least some of the *B. subtilis* proteins are processed and the enzymatic domains released into the culture supernatant similar to what was observed for *S. aureus* LtaS (Ziebandt *et al*., 2001; Gatlin *et al*., 2006; Lu *et al*., 2009). By expressing each of the four *B. subtilis* orthologues in an *S. aureus ltaS* depletion strain it was revealed that LtaS_BS_ and YfnI encode for LTA synthases, capable of producing polyglycerolphosphate polymers (Gründling and Schneewind, 2007a). However, YfnI-produced polymers could not restore the growth defect of an *S. aureus ltaS* depleted strain and had an altered mobility on SDS-PAGE gels, indicative of structural alterations (Gründling and Schneewind, 2007a). No enzyme activity was observed for YqgS or YvgJ. Furthermore, a study on the enzymes in *B. subtilis* revealed that mutants lacking LtaS_BS_ grew slower in PAB medium compared with the wild-type strain and showed a defect in divalent cation homeostasis, an increase in cell chain length and placement of aberrant septa and enhanced cell bending and lysis (Schirner *et al*., 2009). In contrast to *S. aureus*, a *B. subtilis* mutant with disruptions of all four genes could be readily constructed and was viable. However, this mutant showed severe morphological defects and bacteria formed long filaments that spiralled along their long axes (Schirner *et al*., 2009).

To gain further insight into the function of the four *B. subtilis* LtaS-type proteins, we investigated their enzymatic activities using defined *in vitro* and *in vivo* systems. We show that all four orthologues are able to hydrolyse fluorescently labelled PG in a Mn^2+^-dependent manner. Complementation studies using an *S. aureus ltaS* depletion strain revealed that YqgS can produce polyglycerolphosphate polymers, when expressed at sufficiently high levels, suggesting that this protein is also a bona-fide LTA synthase. In contrast, our data indicate that YvgJ functions as an LTA primase, transferring the initial glycerolphosphate subunit onto the glycolipid anchor and therefore producing GroP-Glc_2_-DAG. Furthermore, in *B. subtilis* LtaS_BS_, YfnI or YqgS are sufficient for polyglycerolphosphate polymer production. However, the polymers produced by a strain expressing YfnI in the absence of LtaS_BS_ had an altered mobility on SDS-PAGE gels, presumably caused by an increase in LTA chain length. This result revealed an unexpected interdependency of YfnI enzyme activity on the function of LtaS_BS_ in the natural host *B. subtilis.* Taken together, this study provides the first experimental evidence for the enzymatic activity of all four *B. subtilis* LtaS-type proteins and implications on the LTA synthesis pathway are discussed.

## Results

### All four *B. subtilis*LtaS orthologues are enzymatically active and hydrolyse fluorescently labelled PG

**Fig. 1:**
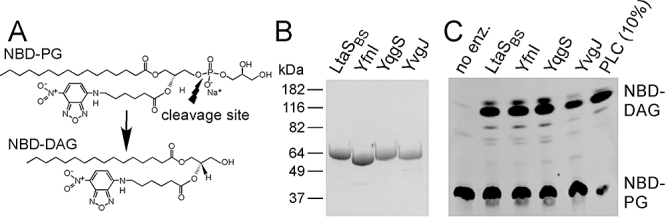
*In vitro* activity of *B. subtilis* LtaS-type enzymes. A. Chemical structures of fluorescently labelled NBD-PG and NBD-DAG lipids with known *S. aureus* LtaS and *B. cereus* PLC cleavage site indicated by an arrow. B. Coomassie stained gel of purified *B. subtilis* LtaS-like proteins. Extracellular enzymatic domains of *B. subtilis* LtaS_BS_, YfnI, YqgS and YvgJ were purified as N-terminal His-tag fusion proteins and 10 µg purified protein separated on a 10% SDS-PAGE gel and visualized by staining with Coomassie brilliant blue. C. TLC analysis of *B. subtilis* LtaS_BS_, YfnI, YvgJ and YqgS *in vitro* reaction products. The NBD-PG lipid substrate was incubated with eLtaS_BS_, eYfnI, eYvgJ or eYqgS enzyme. Subsequently, lipids were extracted and separated by TLC and fluorescent lipid bands visualized by scanning plates with a fluorescence imager. As negative and positive controls, reactions were set up without enzyme or with the *B. cereus* PLC enzyme respectively. Note that only 10% of the PLC reaction was run on the TLC plate. Positions of NBD-PG and presumed NBD-DAG reaction product are indicated on the left and proteins added to each reaction are shown on the top of the panel.

Enzymatic activities have previously only been detected for LtaS_BS_ and YfnI, two of the four *B. subtilis* LtaS orthologues (Gründling and Schneewind, 2007a). Recently, we have developed an *in vitro* assay to measure *S. aureus* eLtaS enzyme activity (Karatsa-Dodgson *et al*., 2010). Using this assay, it was determined that the purified enzymatic domain of *S. aureus* LtaS, eLtaS, but not the active site variant eLtaS-T300A hydrolyses the glycerolphosphate head group of the fluorescently labelled lipid NBD-PG resulting in the production of fluorescently labelled diacylglycerol (NBD-DAG) (Fig. 1A). To determine if the *B. subtilis* LtaS orthologues can perform the same reaction, we cloned, expressed and purified the enzymatic domains of all four proteins as N-terminal His-tagged versions from *E. coli* extracts (Fig. S1 and Fig. 1B). Purified enzymes were mixed with NBD-PG lipid and incubated for 3 h in the presence of MnCl_2_. Subsequently, lipids were extracted, separated by TLC and plates scanned using a fluorescence imager to visualize lipid bands. As a positive control, the commercially available *Bacillus cereus* phospholipase PC-PLC (PLC) was used. This enzyme cleaves PG resulting in the production of DAG (Shinitzky *et al*., 1993). When the reactions were set up using each of the four *B. subtilis* proteins, two major fluorescent lipid bands were observed (Fig. 1C). The faster migrating band had the same mobility as the hydrolysis product produced by PLC, and presumably corresponds to NBD-DAG and the slower migrating band had the mobility of the NBD-PG input lipid. No lipid corresponding to NBD-DAG was detected in reactions set up without enzyme. In summary, these data demonstrate that all four recombinant *B. subtilis* proteins are enzymatically active and suggest that all proteins hydrolyse the phosphodiester bond of NBD-PG resulting in the production of NBD-DAG.

**Fig. 2:**
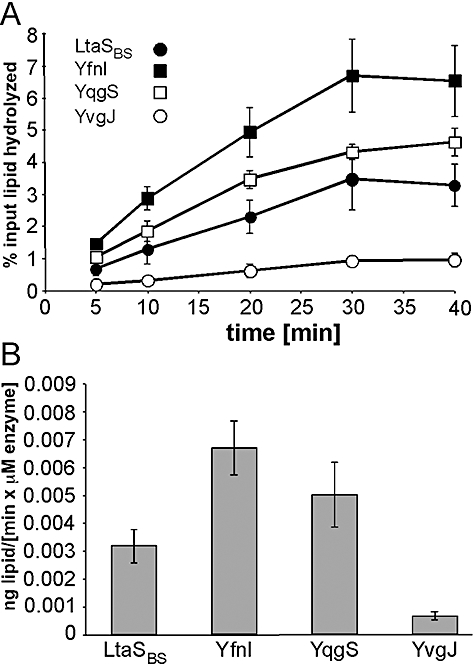
Kinetic measurements for recombinant LtaS_BS_, YfnI, YqgS and YvgJ enzymes. A. Time-course experiment. Enzyme reactions were set up as described under *Experimental procedures*, aliquots removed at the indicated time points and reactions stopped by the addition of chloroform and methanol. Lipids were separated on TLC plates and the NBD-DAG reaction product quantified. For each time point and enzyme the average value and standard deviation of three values is plotted. Three independent experiments were performed and a representative graph is shown. B. Maximal enzyme activity of *B. subtilis* LtaS_BS_, YfnI, YqgS and YvgJ. The slope of the linear fit through the first three data points of the curve shown in (A) was used to calculate the maximal enzyme activity for each *B. subtilis* LtaS orthologue. Three independent time-course experiments were used to determine an average value and standard deviation for the maximal enzyme activity and these values are plotted.

To gain further insight into the relative activity of the four *B. subtilis* proteins, a time-course experiment was performed. Reactions were set up as described above and samples removed at the indicated time points and lipid reaction products analysed (Fig. 2A). The amount of the NBD-DAG reaction product was quantified (Karatsa-Dodgson *et al*., 2010) and % hydrolysis calculated based on the amount of NBD-DAG produced in the PLC control reaction, which proceeds to near completion (Fig. 2A). Three independent experiments were performed and the first three time points, during which the reaction speed appeared to be linear, were used to determine the maximal enzyme activity of the four *B. subtilis* proteins (Fig. 2B). Enzyme activities ranging from 0.0067 ng (YfnI) to 0.0007 ng (YvgJ) lipid hydrolysis/(min × µM enzyme) were measured in our *in vitro* assay set up. These results indicate that while all four enzymes are active, YfnI, LtaS_BS_ and YqgS have > 4.6-fold higher activity as compared with YvgJ.

### All four *B. subtilis*LtaS orthologues are Mn^2*+*^-dependent enzymes with substrate specificity for NBD-PG

**Fig. 3:**
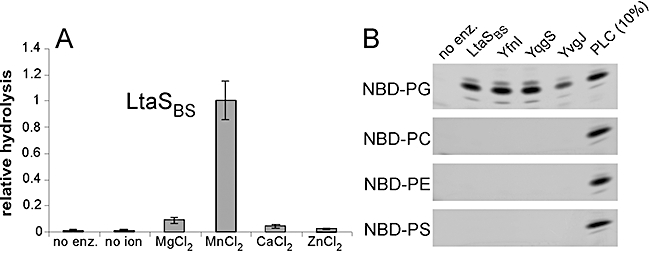
Metal and substrate specificity of recombinant *B. subtilis* LtaS-type enzymes. A. *B. subtilis* LtaS-type enzymes require Mn^2+^ for activity. *In vitro* enzyme assays were set up with NBD-PG lipid as the substrate in the presence of 10 mM MgCl_2_, MnCl_2_, CaCl_2_ or ZnCl_2_ and reactions were initiated by the addition of eLtaS_BS_. As controls, reactions were set up without enzyme or without metal ion added. Samples were incubated for 3 h at 37°C, lipids extracted and separated by TLC. Plates were scanned and signals of the reaction product quantified. Reactions were set up in triplicate and the average value and standard deviation plotted. The average fluorescence reading for the reactions set up with MnCl_2_ was set to 1 and other values were adjusted accordingly. Similar results were obtained for *B. subtilis* YfnI, YqgS and YvgJ (see Fig. S2). B. NBD-PG is the sole lipid substrate for *B. subtilis* LtaS_BS_, YfnI, YqgS and YvgJ. Standard enzyme reactions were set up using NBD-PG, NBD-PS, NBD-PE or NBD-PC as substrate (indicated on the left of the panel) and reactions were initiated by the addition of the different *B. subtilis* enzymes. As a negative control, lipid substrates were incubated without enzyme (no enz.) and as a positive control, a PLC reaction using NBD-PG as substrate was run alongside on each TLC plate in order to determine the mobility of the reaction product. Three independent experiments were performed and a representative result is shown. Note that only the upper part of the TLC plates is shown with the area of the reaction product.

*Staphylococcus aureus* LtaS is a Mn^2+^-dependent metal enzyme (Karatsa-Dodgson *et al*., 2010). In contrast, structural analysis of the soluble enzymatic domain of the *B. subtilis* LtaS_BS_ protein revealed the presence of a Mg^2+^ ion in the active centre (Schirner *et al*., 2009). However, this ion was also present in the crystallization buffer and as such may not reflect the ion relevant for enzyme activity. To test which metal ion is required for the activity of the *B. subtilis* enzymes, *in vitro* assays were set up in the presence of different divalent metal ions and the signal for the reaction product quantified. As presented in Fig. 3A and Fig. S2, all four proteins showed the highest activity in the presence of MnCl_2_. Addition of MgCl_2_ and CaCl_2_ in place of MnCl_2_ resulted only in weak enzyme activity and no activity above background was seen in the presence of ZnCl_2_.

*Staphylococcus aureus* LtaS uses NBD-labelled PG lipid as substrate but not NBD-PC, NBD-PS or NBD-PE (Karatsa-Dodgson *et al*., 2010). Next, we tested the substrate specificity of the four *B. subtilis* enzymes and found that also these enzymes use only NBD-PG as substrate and not NBD-PC, NBD-PS or NBD-PE (Fig. 2B). Taken together, these results strengthen the hypothesis that all members of the LTA synthase enzyme family are Mn^2+^-dependent metal enzymes that only use lipids with a glycerolphosphate head group as substrate.

### *B. subtilis*YqgS is an LTA synthase, capable of producing polyglycerolphosphate chains

**Fig. 4:**
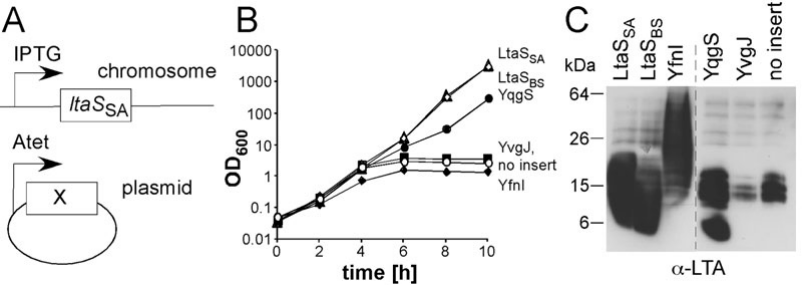
Functional complementation of an *S. aureus ltaS*-depletion strain with *B. subtilis ltaS_BS_, yfnI, yqgS* or *yvgJ* expressed from a multicopy plasmid. A. Schematic representation of complementation strains. *S. aureus* strains used for complementation analysis contain the chromosomal copy of *ltaS* under IPTG inducible expression control and harbour a multicopy plasmid (pCN34) for expression of LtaS orthologues from the tetracycline inducible promoter. B. Bacterial growth curves. Washed overnight cultures of *S. aureus* strains ANG1571 (LtaS_SA_-expressing), ANG1662 (LtaS_BS_-expressing), ANG1573 (YfnI-expressing), ANG1654 (YqgS-expressing) (ANG1658) (YvgJ-expression) and ANG1130 (containing empty vector pCN34; no insert as negative control) were diluted 1:100 into fresh medium containing 300 ng ml^−1^ Atet and growth was monitored by determining OD_600_ readings at the indicated time points. All cultures were back-diluted 1:100 at the 4 h time point and cultures with strains expressing LtaS_SA_, LtaS_BS_ and YqgS were back diluted a second time at the 8 h time point to maintain cultures in the logarithmic growth phase. C. LTA detection by Western blot. The same *S. aureus* strains and growth conditions as described above were used for LTA analysis by Western blot. At the 4 h time point, 1 ml culture aliquots were removed and samples prepared and analysed by Western blot as described in the *Experimental procedures* section. For LTA detection, the mouse monoclonal LTA antibody (Clone 55 from Hycult biotechnology) and the HRP-conjugated anti-mouse IgG antibody (Cell Signaling Technologies, USA) were used at 1:5000 and 1:10 000 dilutions respectively. Sizes of proteins standards run in parallel are shown on the left of the panel and proteins expressed in each strain are given above each lane. Note that the LTA Western blot with samples isolated from *S. aureus* strains expressing YqgS, YvgJ or containing the empty vector (no insert) was exposed four times longer.

The finding that YqgS and YvgJ can cleave NBD-PG is somewhat in contrast to a previously performed complementation analysis that showed that YqgS and YvgJ could not promote LTA synthesis in *S. aureus* (Gründling and Schneewind, 2007a). One reason why no *in vivo* activity for YqgS and YvgJ was observed could be insufficient expression achieved from the single-copy integration vector used in the previously performed complementation study (Gründling and Schneewind, 2007a). To test whether expression of YqgS and YvgJ from a multicopy plasmid would reveal an *in vivo* enzyme function for these proteins, all four *B. subtilis* genes coding for LtaS-type proteins and the *S. aureus ltaS* gene were cloned under tetracycline-inducible promoter control into the multicopy plasmid vector pCN34 (Fig. 4A). In addition, the ribosome binding site (RBS) of *yqgS* was replaced with the RBS that precedes the *S. aureus ltaS* gene, in which a string of Gs is located eight bases in front of the ATG start codon, indicative of a good RBS (Vellanoweth and Rabinowitz, 1992). Resulting plasmids and the empty pCN34 vector control were introduced into *S. aureus* strain ANG499, which carries the chromosomal copy of *ltaS* under IPTG inducible *spac* promoter control (Fig. 4A). Functional complementation of *ltaS* was examined in the resulting strains after removal of IPTG by the addition of anhydrotetracycline to the growth medium for expression of the different *B. subtilis* LtaS orthologues. As described previously, LtaS_BS_ was able to complement both growth and LTA production in the *S. aureus ltaS* depletion strain whereas YfnI could only promote polyglycerolphosphate synthesis but not the growth of *S. aureus* (Fig. 4B and C). Interestingly, expression of YqgS under these conditions could also restore bacterial growth, albeit to a lesser extent than LtaS_BS_. Furthermore, upon increasing the exposure time of Western blots, a YqgS-depended signal for a faster migrating polyglycerolphosphate polymer was detected, next to other bands, which were also seen in the negative (no insert) control sample (Fig. 4C). We assume that these other bands also present in the negative control sample are LTA-specific (as they are detected with a monoclonal antibody) and either due to a slight leakiness of the inducible spac promoter system or due to small amounts of LTA remaining on the cells even 4 h after the shut down of LtaS expression as these bands are absent from samples isolated from an *S. aureus* strain with a complete *ltaS* deletion (R. Corrigan, unpublished results). In contrast, even under these conditions, YvgJ expression did not result in growth of *S. aureus* or LTA production. Taken together, these results suggest that YqgS functions as an LTA synthase, capable of promoting polyglycerolphosphate LTA backbone synthesis.

### *B. subtilis*YvgJ functions as an LTA primase

**Fig. 5:**
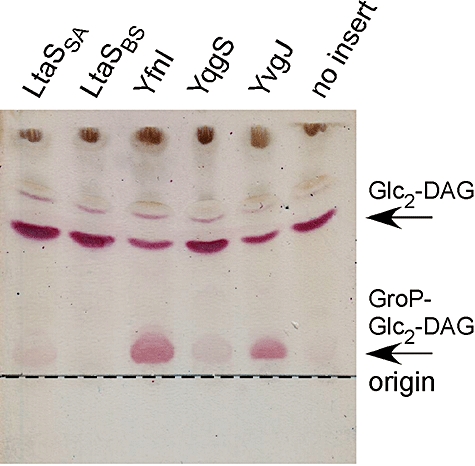
TLC analysis of glycolipids. *S. aureus* strains ANG1571 (LtaS_SA_-expressing), ANG1662 (LtaS_BS_-expressing), ANG1573 (YfnI-expressing), ANG1654 (YqgS-expressing), ANG1658 (YvgJ-expressing) and ANG1130 (containing empty vector pCN34, no insert as negative control) were grown to mid-log phase and lipids extracted as described in the *Experimental procedures* section. Five hundred µg total membrane lipids were separated by TLC and glycolipids visualized by staining with α-naphthol/sulphuric acid. The position of the origin is indicated by a dashed line, positions of presumed Glc_2_-DAG (top band) and GroP-Glc_2_-DAG (bottom band) lipids are marked with arrows on the right of the panel and proteins expressed in the different strains are indicated above each lane.

**Fig. 6:**
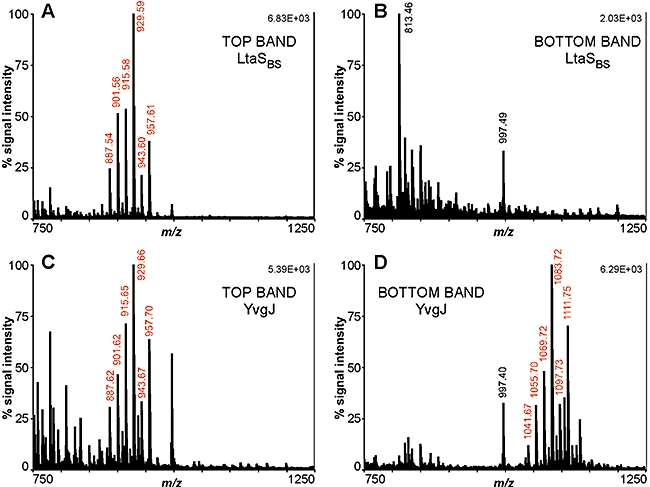
MALDI-TOF analysis of glycolipids produced by LtaS_BS_ and YvgJ-expressing *S. aureus* strains. A total of 2.5 mg lipids isolated from LtaS_BS_ or YvgJ-expressing *S. aureus* strains were separated by TLC and lipids corresponding to top and bottom glycolipids bands extracted and analysed by MALDI-TOF mass spectrometry. Spectra were recorded in the reflector positive ion mode and are shown for (A) LtaS_BS_ top band, (B) LtaS_BS_ bottom band, (C) YvgJ top band and (D) YvgJ bottom band. Maximal signal intensity is shown in the top right corner in each panel. Note that the maximum signal intensity in panel B is lower than in the other three panels, which amplifies background signals in the normalized representation shown. Observed masses corresponding to calculated masses of glycolipids are shown in red. Three independent experiments were performed and representative spectra are shown.

Expression of *yvgJ* from a strong promoter and a multicopy plasmid did not restore growth or LTA production in the *S. aureus ltaS* depletion strain, indicating that YvgJ may not function as an LTA synthase. In a previous study, it has been shown that the *Listeria monocytogenes* LtaS-type protein Lmo0644 encodes for an enzyme that can transfer one glycerolphosphate subunit onto the glycolipid anchor and hence we termed this enzyme LTA primase (Webb *et al*., 2009). In *S. aureus* and *B. subtilis* this reaction would lead to the production of the GroP-Glc_2_-DAG. To test if YvgJ could function as an LTA primase and to investigate if any of the other *B. subtilis* orthologues are involved in the production of glycolipid intermediates, membrane lipids were extracted and analysed from *S. aureus* strains expressing *B. subtilis* LtaS_BS_, YfnI, YqgS, YvgJ or *S. aureus* LtaS_SA_ as a control. Five hundred micrograms purified lipids extracted from log-phase cultures were separated on TLC plates and glycolipids visualized by staining with α-naphthol/H_2_SO_4_ (Fig. 5). The LTA glycolipid anchor Glc_2_-DAG (Top band; see also mass spectrometry analysis below) could be detected in all samples. However, the intensity of this glycolipid band was reduced in samples isolated from YfnI and YvgJ expressing strains and a concomitant accumulation of the lower glycolipid band was observed. This lipid species had the mobility as expected for a GroP-di-saccaride-DAG lipid (Webb *et al*., 2009), which would be consistent with the accumulation of the GroP-Glc_2_-DAG intermediate. To provide further experimental evidence for this notion, lipids from *S. aureus* strains expressing LtaS_BS_ (predominantly producing the top glycolipid band) and YvgJ (accumulating the bottom glycolipid band) were separated by TLC and lipids corresponding to α-naphthol/H_2_SO_4_ positive areas extracted and analysed by MALDI TOF mass spectrometry, which was performed as described previously (Webb *et al*., 2009). Sodium adducts of the glycolipids Glc_2_-DAG and GroP-Glc_2_-DAG with C15 and C18 acyl-chains have an absolute calculated mass of 929.62 and 1083.72 respectively (see Table 1). In agreement with these expected masses, m/z signals of 929.59 and 929.66 were observed for lipids isolated from the top bands of samples obtained from LtaS_BS_ and YvgJ expressing strains respectively (Fig. 6A and C). In addition, a strong mass signal of 1083.73 as expected for GroP-Glc_2_-DAG was obtained for lipids isolated from the bottom band of the YvgJ-expressing strain (Fig. 6D). A corresponding signal was absent from samples prepared from the LtaS_BS_ expressing strains (Fig. 6B), which does not show an accumulation of this glycolipid. A complete list of predicted and observed masses for glycolipids Glc_2_-DAG and GroP-Glc_2_-DAG with varying acyl-chain length is given in Table 1. Taken together, these data suggest that YvgJ functions *in vivo* as an LTA primase capable of transferring the initial glycerolphosphate subunit onto the glycolipid anchor producing GroP-Glc_2_-DAG. Furthermore, despite the fact that YfnI acts as an LTA synthase, it also appears to be very efficient in synthesizing the GroP-Glc_2_-DAG intermediate.

**Table 1:** Predicted and observed masses of glycolipids isolated from membranes of *S. aureus* strains expressing *B. subtilis* LtaS_BS_ or YvgJ

Possible fatty acid chain length	Chemical formula	Predicted mass	Observed mass – LtaS_BS_	Observed mass – YvgJ
Top band: Glc_2_-DAG				
C15/C15	C_45_H_84_Na_1_O_15_	887.57	887.54	887.62
C15/C16	C_46_H_86_Na_1_O_15_	901.59	901.56	901.62
C15/C17	C_47_H_88_Na_1_O_15_	915.60	915.58	915.65
C15/C18	C_48_H_90_Na_1_O_15_	929.62	929.59	929.66
C16/C18	C_49_H_92_Na_1_O_15_	943.63	943.60	943.67
C17/C18	C_50_H_94_Na_1_O_15_	957.65	957.61	957.70
Bottom band: GroP-Glc_2_-DAG				
C15/C15	C_48_H_91_Na_1_O_20_P_1_	1041.57	Absent	1041.67
C15/C16	C_49_H_93_Na_1_O_20_P_1_	1055.59	Absent	1055.70
C15/C17	C_50_H_95_Na_1_O_20_P_1_	1069.61	Absent	1069.72
C15/C18	C_51_H_97_Na_1_O_20_P_1_	1083.62	Absent	1083.72
C16/C18	C_52_H_99_Na_1_O_20_P_1_	1097.64	Absent	1097.73
C17/C18	C_53_H_101_Na_1_O_20_P_1_	1111.65	Absent	1111.75

### Contribution of LtaS_BS_, YfnI, YqgS and YvgJ to LTA synthesis in *B. subtilis*

**Fig. 7:**
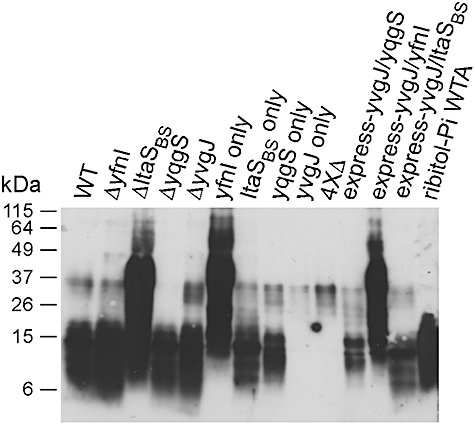
LTA production by wild-type and mutant *B. subtilis* strains. Samples for LTA analysis by Western blot were prepared from overnight cultures of wild-type and mutant *B. subtilis* 168 strains and from a *B. subtilis* 168 hybrid strain expressing ribitolphosphate wall teichoic acid. Samples were separated on a 15% SDS-PAGE gel, transferred to a PVDF membrane and LTA detected by Western blot using the humanized monoclonal LTA-specific antibody (Biosynexus Incorporated) and the HRP-linked anti-human antibody (DakoCytomation) at 1:10 000 dilutions. Sizes of protein standards run in parallel are indicated on the left of the panel and strains used are indicated above each lane, with abbreviations given in strain Table 2.

A *B. subtilis* strain deleted of all four *ltaS*-like genes has been constructed previously and is viable (Schirner *et al*., 2009). Several phenotypes were associated with the deletion of these genes; a single *ltaS_BS_* mutant formed chains, *ltaS_BS_*/*yqgS* and *ltaS_BS_*/*yvgJ* double mutants had sporulation defects and cells lacking all four genes formed long chains and spiraled along their long axis (Schirner *et al*., 2009). To correlate deletions of *ltaS*-like genes with the cellular LTA content, we created single, triple mutants and the quadruple mutant in the *B. subtilis* strain 168 by replacing respective genes with antibiotic resistance markers. Mutations and strains were confirmed by PCR analysis and appropriate antibiotic resistance pattern (*yfnI::Cam; yflE::Kan; yqgS::Spec; yvgJ::Erm*) (Fig. S3). In addition, when mutant strains were grown in PAB medium either overnight to stationary phase or until mid-log phase, we observed similar morphological alterations as previously described (Fig. S4) (Schirner *et al*., 2009). The quadruple mutant formed filaments that spiraled along their axes and the *ltaS_BS_* mutant formed filaments primarily during exponential growth (Fig. S4). Next, cell extracts were prepared from overnight cultures of wild-type and mutant *B. subtilis* strains and the LTA content analysed by Western blot. Initially, we attempted to use the mouse monoclonal LTA antibody, which was used for the above-described *S. aureus* experiments. However, when we used this antibody the Western blot signal was not strong enough to detect LTA from *B. subtilis*. We speculate that this could either be due to lower LTA amounts or the additional sugar modifications present on the LTA backbone in *B. subtilis*. However, when we used a humanized monoclonal LTA-specific antibody, which is supplied at a higher concentration, an LTA-specific signal was obtained for a sample isolated from the wild-type *B. subtilis* 168 strain (WT) (Fig. 7). This signal was absent from samples isolated from a *B. subtilis* strain lacking all four *ltaS*-like genes (4xΔ) or a strain expressing YvgJ-only (Fig. 7). Of note, in several samples including the YvgJ-only and 4xΔ samples, an additional signal in the 30 kDa area was observed, which we assume is unrelated to LTA and could be cross-reactivity towards the WTA polymer, which in *B. subtilis* 168 is also predominantly made up of glycerolphosphate subunits. This signal was less abundant in the *B. subtilis* 168 hybrid strain L5703 (Karamata *et al*., 1987) expressing ribitolphosphate WTA (ribitol-Pi WTA) in place of glycerolphosphate WTA (Fig. 7). Deletion of *yfnI*, *yqgS* or *yvgJ* alone did not significantly affect LTA production. Interestingly, deletion of *ltaS_BS_* resulted in the production of LTA with an altered mobility on SDS-PAGE gels, indicative of structural changes (see below). The production of this altered LTA was attributed to the function of YfnI, as a *B. subtilis* strain expressing YfnI-only showed a similar altered LTA profile (Fig. 7). This also indicates that LtaS_BS_ affects the activity of YfnI, revealing an unexpected enzymatic interdependence of the activity of two LTA-synthases in *B. subtilis*.

In the case of *L. monocytogenes*, which produces an LTA primase and one LTA synthase, a clear difference in LTA production was seen when the LTA primase was inactivated (Webb *et al*., 2009). In *B. subtilis* no obvious difference in LTA production was observed upon inactivation of the LTA primase YvgJ (Fig. 7; compare LTA profile of Δ*yvgJ* strain with wild-type strain). To test more specifically if the *B. subtilis* YvgJ enzyme works together with one of the LTA synthases, we created three *B. subtilis* double mutant strain in which YvgJ is expressed with one of the LTA synthase enzyme LtaS_BS_, YfnI or YqgS and compared LTA production in these strains with a strain, which expresses the synthase alone. No difference in LTA production was observed for any of the LTA synthases in the absence of the LTA primase (Fig. 7; compare lanes LtaS*_BS_*-only with express-YvgJ/LtaS_BS_; YfnI-only with express-YvgJ/YfnI or YqgS-only with express-YvgJ/YqgS). This indicates that in contrast to *L. monocytogenes* all *B. subtilis* LTA synthases can efficiently initiate LTA production even in the absence of a dedicated LTA primase. However, as shown above and further analysed below, in *B. subtilis* LtaS_BS_ affects the function of YfnI.

### YfnI synthesizes glycerolphosphate polymers of increased length

**Fig. 8:**
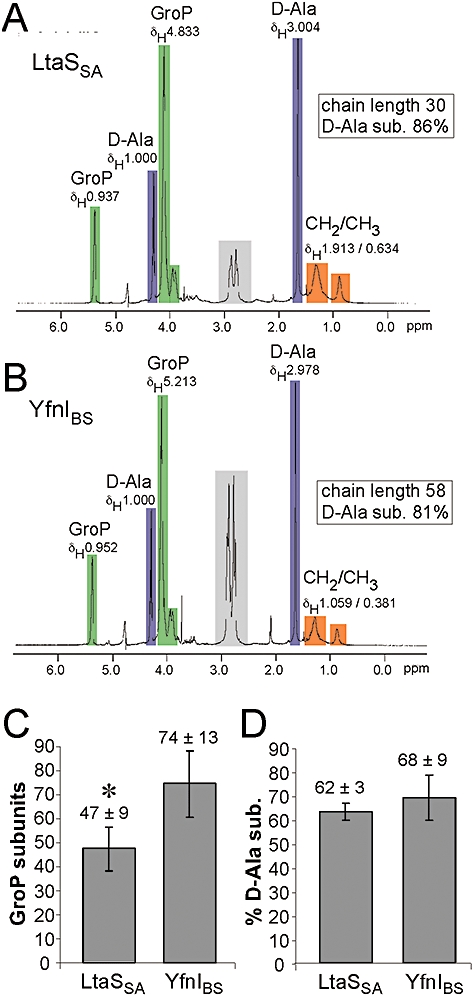
NMR and biochemical analysis of purified LTA. A and B. NMR analysis. Large cultures of *S. aureus* strains (A) ANG514 (LtaS_SA_-expressing) and (B) ANG515 (YfnI-expressing) were grown and LTA purified as described in the *Experimental procedures* section. One milligram purified LTA was suspended and lyophilized several times in D_2_O to exchange ^1^H for ^2^H deuterons and ^1^H NMR spectra were recorded at 600 MHz, 300 K. The signals derived from citrate, a buffer component used during LTA purification and retained in the samples are marked in grey. The different signals previously assigned to LTA components (Morath *et al*., 2002) are colour coded [blue – D-Ala (4 protons per D-Ala group), green – GroP (5 protons per GroP group), orange – CH_2_/CH_3_ groups of fatty acids (59 protons per lipid anchor)]. The integration values are shown above each signal. Chain length was determined by calculating the ratio of integral values for GroP to CH_2_/CH_3_ groups in fatty acids and % D-Ala substitution by calculating the ratio of integral values for D-Ala to GroP x 100 and taking into account the number of protons for each signal. NMR analysis was performed on four independently isolated LTA samples for each strain and a representative result is shown. C and D. Biochemical analysis of LTA. LTA extracted from strains ANG514 (LtaS_SA_) and ANG515 (YfnI_BS_) was subjected to a biochemical analysis. Phosphate, glucose and D-Ala contents were determined as described in the *Experimental procedures* section. GroP, D-Ala and glucose solutions of known concentrations were used as standards. The chain length in GroP subunits (C) was determined by calculation of the ratio of phosphate/½ glucose concentration and the % D-Ala substitution (D) by calculating the ratio of D-Ala/phosphate concentration × 100. Biochemical analysis was performed on four independently isolated LTA samples for each strain and the mean and standard deviation is shown. The difference in chain length is statistically significant and indicated with an asterisk (*) (two-tailed *P*-value of 0.017; unpaired *T*-test) while the difference in D-Ala modification is not (two-tailed *P*-value of 0.355, unpaired *T*-test).

Polymers produced by YfnI in the absence of LtaS_BS_ migrate with a slower mobility on SDS-PAGE gels, both when synthesized in the natural host *B. subtilis* (Fig. 7) or in the heterologous host *S. aureus* (Fig. 4C; Gründling and Schneewind, 2007a). To gain insight into structural alterations of polymers synthesized by YfnI and to provide further information on the enzymatic activity of this protein, we isolated LTA from an *S. aureus* YfnI-expressing strain and compared its composition with polymers produced by LtaS. We chose the YfnI-expressing *S. aureus* strain for this analysis as we have methods established to isolate LTA from this organism and, based on Western blot analysis, it appeared that *S. aureus* produces larger amounts of LTA as compared with *B. subtilis*. LTA was isolated from mid-log cultures of *S. aureus* strains ANG514 (LtaS-expressing) and ANG515 (YfnI-expressing) using a 1-butanol extraction method and purified by hydrophobic interaction chromatography. LTA was purified from four independently grown cultures for each strain and analysed by nuclear magnetic resonance (NMR) and standard biochemical assays (Fig. 8). Representative NMR spectra are shown in Fig. 8A and B. Based on the NMR analysis of all four independently isolated LTA samples, an average glycerolphosphate chain length of 35 ± 6 (LtaS) and 54 ± 6 (YfnI) and average D-Ala modification of 82 ± 5% (LtaS) and 74 ± 6% (YfnI) was calculated. The difference in chain length is considered to be statistically significant (*P*-value = 0.0048), while the difference in D-Ala modifications is not quite statistically significant (*P*-value = 0.083). In addition, standard biochemical assays were used to determine the phosphate, glucose and D-Ala content in the purified LTA samples (Schnitger *et al*., 1959; Kunst *et al*., 1984; Grassl and Supp, 1995). LTA in a wild-type *S. aureus* strain is linked nearly exclusively to the glycolipid anchor Glc_2_-DAG (Duckworth *et al*., 1975) and hence the ratio of the phosphate concentration per two glucose molecules can be used for the chain length determination and the ratio of D-Ala to phosphate concentration gives a measure for % d-alanylation. Applying these calculations to LtaS- or YfnI-produced polymers, revealed an average chain length of 47 ± 9 and 74 ± 14 (Fig. 8C) and % d-alanylation of 62 ± 4 and 68 ± 9 (Fig. 8D) respectively. This biochemical analysis gives a slightly longer chain length for LtaS and YfnI-produced LTA as compared with the NMR analysis. However, both methods indicate that YfnI-produced polymers are significantly longer; 1.5× based on NMR or 1.6× based on biochemical assays than LtaS-produced polymer, but remain linked to a glycolipid anchor. On the other hand there does not appear to be a statistically significant difference in the amount of D-Ala substitutions. While this analysis was performed in *S. aureus*, we speculate that the observed mobility shift of YfnI-produced polymers in the natural host *B. subtilis* is also due to an increase in chain length, suggesting that in the absence of LtaS, YfnI-becomes more efficient in LTA synthesis.

## Discussion

Polyglycerolphosphate LTA is found in the cell envelope of many Gram-positive bacteria. In bacteria that belong to the phylum Firmicutes, the backbone of this polymer is synthesized by the LTA synthase enzyme LtaS (Gründling and Schneewind, 2007a; Rahman *et al*., 2009). In contrast to *S. aureus*, which produces a single LTA synthase, *B. subtilis* encodes four LtaS-like proteins, namely LtaS_BS_, YfnI, YqgS and YvgJ (Gründling and Schneewind, 2007a; Schirner *et al*., 2009). Here, we report on the enzymatic activities of these four *B. subtilis* LtaS-like proteins (Figs 1 and 2) and show that LtaS_BS_, YfnI and YqgS are bona-fide LTA synthases that can synthesize polyglycerolphosphate polymers (Figs 4C and 7). In contrast, YvgJ is an LTA primase, which uses the glycerolphosphate head group of the membrane lipid PG to form the glycolipid GroP-Glc_2_-DAG (Figs 5 and 6), which is assumed to be an LTA synthesis intermediate.

It is interesting to note that the three recombinant LTA synthases (LtaS_BS_, YfnI and YqgS) showed > 4.5-fold higher activity in the *in vitro* assay system as compared with the enzymatic domain of the LTA primase YvgJ (Fig. 2). A similar observation was made with recombinant versions of the *L. monocytogenes* LTA synthase and LTA primase (Karatsa-Dodgson *et al*., 2010), indicating that there may be a correlation between the activity of these enzymes and their ability to produce actual glycerolphosphate polymers. Other general features revealed through the use of the *in vitro* assay system were that LTA sythases and primases require Mn^2+^ for *in vitro* enzyme activity and only seem to accept lipids with a glycerolphosphate head group as substrate but not lipids with other head groups such as PC, PE or PS (Fig. 3).

**Fig. 9:**
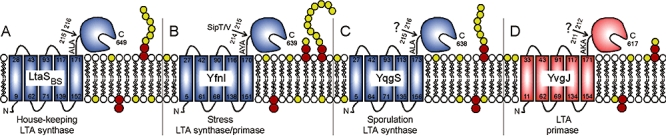
Schematic representation of *in vivo* activities of the four *B. subtilis* LtaS-type enzymes. A. *B. subtilis* LtaS_BS_ is the ‘house-keeping’ LTA synthase, which is active during vegetative growth. B. *B. subtilis* YfnI is assumed to be the ‘stress’ LTA synthase as *yfnI* transcription is controlled by sigma M, which is important during cell envelope stress. YfnI is capable of promoting polyglycerolphosphate synthesis as well as producing the GroP-Glc_2_-DAG glycolipid intermediate. Here we show that YfnI activity is influenced by the presence/absence of LtaS_BS_. Processed forms of both, LtaS_BS_ and YfnI have been detected in the culture supernatant and processing of YfnI is reduced in the combined absence of the two signal peptidases SipT and SipV (Antelmann *et al*., 2001). C. *B. subtilis* YqgS has LTA synthase activity and is important during the sporulation process.D. YvgJ functions as an LTA primase synthesizing the glycolipid intermediate GroP-Glc_2_-DAG. Although YqgS and YvgJ contain an AXA motif it is not clear if these enzymes are processed in *B. subtilis*. LTA synthases are depicted in blue and LTA primases in red. Numbers refer to amino acid positions and arrows indicate cleavage or potential cleavage sites.

Two different models have been proposed for LTA biosynthesis that differ in the enzyme activity, which is required for the actual linkage of the glycerolphosphate polymer to the glycolipid anchor (recently reviewed in Rahman *et al*., 2009). For one model, it was proposed that an ‘LTA transferase’ moves fully synthesized polyglycerolphosphate polymers from a DAG lipid anchor onto a glycolipid anchor. This was based on the following observations: In *Streptococcus sanguis* a significant amount of polyglycerolphosphate ‘intermediates’ linked to DAG are present in the membrane (Chiu *et al*., 1993). In the absence of glycolipids due to mutations in genes necessary for their synthesis (Button and Hemmings, 1976; Kiriukhin *et al*., 2001; Fedtke *et al*., 2007) or natural lack of these genes as found in some *Bacillus* sp. (Iwasaki *et al*., 1986), polyglycerolphosphate polymers are directly linked to DAG. Hence, it was proposed that the DAG-linked polymers are natural LTA synthesis intermediates, which are subsequently moved by an LTA transferase enzyme onto the glycolipid anchor. In the second model an LTA primase adds the first glycerolphosphate subunit to the glycolipid anchor to form the GroP-glycolipid intermediate. Subsequently, an LTA synthase adds additional glycerolphosphate subunits onto this GroP-glycolipid intermediate to produce the LTA backbone chain. The discovery of an LTA primase in *L. monocytogenes* (Webb *et al*., 2009) and, as described in this study, now also in *B. subtilis* favours the second model. However, we postulate that the action of an LTA primase and the production of the GroP-glycolipid intermediate will only in some cases and to some extent aid in the LTA synthesis process. We propose, in addition to this two-enzyme system, a slightly altered version of the latter model in which only a single enzyme is needed for LTA synthesis. In this model, a single enzyme can directly start and extend the glycerolphosphate chain on the glycolipid anchor, regardless of how many LtaS-type enzymes are encoded in the genome. We have shown that enzymes with LTA synthase and LTA primase activity belong to the same family of proteins (LtaS-type enzymes). Members of this protein family show a high degree of identity on the amino acid level and have the same predicted membrane topology and domain structure (Fig. 9). Therefore, we are confident that in the genome of *S. aureus* and several other Gram-positive bacteria, only one LtaS-type enzyme is encoded and hence we suggest that this enzyme functions as both an LTA synthase and an LTA primase. In addition, the *B. subtilis* YfnI enzyme produces both polyglycerolphosphate polymers and the GroP-Glc_2_-DAG intermediate (Figs 4C, 5 and 7), providing further evidence that the same LtaS-type enzyme can be an LTA synthase and an LTA primase. Furthermore, deletion of the dedicated LTA primase YvgJ in *B. subtilis* does not lead to an obvious difference in LTA production, suggesting that all *B. subtilis* LTA synthases are able to efficiently initiate LTA synthesis independent of the activity of a dedicated LTA primase (Fig. 7). Therefore, we suggest that while LtaS-type enzymes are very selective for their lipid substrate (they can only cleave the head group of PG), at least some of them have a relaxed specificity towards the acceptor lipid that can be used for the subsequent glycerolphosphate transfer reaction. For example, *S. aureus* LtaS can use DAG, Glc_2_-DAG, GroP-Glc_2_-DAG and the polyglycerolphosphate chain. However, the efficiencies with which these different acceptor molecules can be used will vary between each individual enzyme and dictate how efficiently LTA can be synthesized in the absence of glycolipids or a dedicated primase and might also influence the final chain length of LTA molecules.

Using blast homology searches, we investigated whether it is possible to distinguish between LTA synthases and dedicated LTA primases (such as the *B. subtilis* YvgJ and *L. monocytogenes* Lmo0644 proteins). However, we did not find any motifs that would allow us to predict which LtaS-type enzyme is an LTA synthase and which protein would only function as an LTA primase. Additional structural information on LTA synthases and LTA primases especially in their full-length membrane form, combined with additional *in vitro* assay studies investigating specifically the glycerolphosphate transfer reaction in the presence of different acceptor molecules, would help us shed light on this question.

*Bacillus subtilis* strains lacking individual *ltaS*-like genes or combinations of the four genes display several phenotypes. For instance, a single *ltaS_BS_* mutant formed chains, an *ltaS_BS_*/*yqgS* double mutant has a sporulation defect and cells lacking all four genes form long chains and spiraled along their long axis (Fig. S4) (Schirner *et al*., 2009). Here, we show that the mere presence or absence of polyglycerolphosphate polymers cannot account for the observed filamentation and sporulation defect, as a strain deleted for *ltaS_BS_* or *ltaS_BS_*/*yqgS* is still capable of producing a glycerolphosphate polymer (Fig. 7). Previously, both LtaS_BS_ and YqgS expressed as GFP-fusion proteins from an inducible promoter system were found to localize preferentially to the division site or sporulation septum (Schirner *et al*., 2009). Taken together with our finding on the LTA production in the different mutant strains (Fig. 7), this would indicate that in the absence of LtaS_BS_, YqgS produces functional polyglycerolphosphate polymers at the sporulation septum and that polymers synthesized by YfnI are either not produced at the sporulation septum or are not functional due to their structural alterations (see below).

We show that a *B. subtilis ltaS_BS_* deletion strain as well as a strain expressing YfnI as a sole LTA synthase produces polymers that migrated slower on SDS-PAGE gels compared with wild-type LTA (Fig. 7). This was also observed when *yfnI* was expressed in an *S. aureus ltaS* depletion strain (Fig. 4C) (Gründling and Schneewind, 2007a), indicating that the altered mobility of LTA produced by YfnI in *S. aureus* is not an artefact, but reflects the natural property of this enzyme. NMR and biochemical analysis of LTA purified from *S. aureus* strains expressing LtaS or YfnI revealed a 1.5- to 1.6-fold increase in chain length for YfnI-produced polymers (Fig. 8), and this presumably results in the slower mobility on SDS-PAGE gels. The presence of longer polymers in the absence of LtaS_BS_ is somewhat puzzling and suggests that the activity of YfnI changes in the presence of LtaS_BS_, or that YfnI and LtaS_BS_ compete for the PG lipid substrate and that YfnI can only synthesize long polymers in the absence of LtaS_BS_ due to an increased availability of PG. Alternatively, LtaS_BS_ could trim YfnI-produced polymers and hence these long polymers are only seen in the absence of LtaS_BS_.

Currently, it is not known if there are any natural conditions under which YfnI would be expressed in the absence of LtaS_BS_ and hence lead to the production of elongated LTA molecules. A previous study using transcriptional *lacZ* reporter gene fusions showed low expression of *yqgS*, *yvgJ* and *yfnI* compared with *ltaS_BS_* during growth of *B. subtilis* in PAB medium (Schirner *et al*., 2009), which, together with all other evidence, suggests that LtaS_BS_ is the ‘house-keeping’ LTA synthase. In proteomic studies processed forms of both LtaS_BS_ and YfnI were detected in the culture supernatant of late-exponential phase *B. subtilis* cultures when grown in minimal medium (Hirose *et al*., 2000), showing that both of these LTA synthases are produced under these conditions. In addition, YfnI was found in culture supernatants of exponential as well as stationary phase *B. subtilis* cultures when grown in L-broth (Antelmann *et al*., 2001). But most notably, it has also been described that *yfnI* expression is controlled by the alternative sigma factor sigma M (Jervis *et al*., 2007; Eiamphungporn and Helmann, 2008) and hence its expression is activated under specific stress conditions such as high salt, low pH, heat and certain antibiotics (Thackray and Moir, 2003; Jervis *et al*., 2007; Eiamphungporn and Helmann, 2008). It could be that under these conditions LTA of slightly different structure is synthesized to better cope with specific stress conditions. However, it has been reported that a *yfnI* deletion strain is not more sensitive to salt stress as compared with a wild-type strain (Schirner *et al*., 2009), but additional studies are needed to determine if YfnI could indeed be a ‘stress LTA-synthase’. During sporulation YqgS is essential in the absence of the ‘house-keeping’ LTA synthase LtaS_BS_ (Schirner *et al*., 2009), and therefore could be termed sporulation LTA synthase (Fig. 9). The function of the LTA primase YvgJ during growth of *B. subtilis* is least clear and only the somewhat reduced sporulation efficiency in the absence of both LtaS_BS_ and YvgJ would indicate that the GroP-Glc_2_-DAG intermediate produced by YvgJ plays a role during the sporulation process (Fig. 9). It is interesting to note that accumulation of the GroP-Glc_2_-DAG glycolipid intermediate upon YvgJ expression leads to a concomitant decrease in the amount of the glycolipid Glc_2_-DAG (Fig. 5), suggesting that the total glycolipid pool is held constant in the cell and that GroP-Glc_2_-DAG is part of this glycolipid pool. This could indicate that GroP-Glc_2_-DAG might still be able to traverse the membrane and reach the cytoplasm of the cell where it could cause a feedback inhibition on cytoplasmic glycolipid synthesizing enzymes (UgtP in *B. subtilis* or YpfP in *S. aureus*).

Most *Bacillus* species produce LTA of the polyglycerolphosphate type (Iwasaki *et al*., 1986; 1989;). However, based on literature searches and sequence analysis, at least *Bacillus circulans*, *Bacillus pseudofirmus* OF4 and *Bacillus halodurans* C125 lack the LTA polymer or LtaS-type enzymes. *B. circulans* falls into an ungrouped class of *Bacillaceae* species (Xu and Cote, 2003) and the latter two strains are alkaliphilic bacteria (Iwasaki *et al*., 1989; Takami *et al*., 2000). It has been shown that the alkaliphilic strain *B. halodurans* contains, in place of teichoic acids, teichuronopeptides as major cell wall components, which are co-polymers of polyglutamic acid and polyglucoronic acid (Takami *et al*., 2000). Several sequenced *Bacillus* species, namely *Bacillus selenitireducen*s MLS10, *Bacillus coagulans* 36D1 and *Bacillus coahuilensis* m4-4, apparently encode only a single LtaS homologue, which should be sufficient for polyglycerolphosphate LTA synthesis. However, the majority of *Bacillus* species encode multiple LtaS-type proteins. At the present it is still not completely understood why bacteria such as *B. subtilis* produce multiple proteins. However as shown here, all four proteins are involved in the LTA synthesis process, they have distinct enzymatic activities within the cell and there is a functional interdependency of their enzymatic activities. Presumably the co-ordinate expression and activity of these proteins allow *B. subtilis* to fine-tune LTA synthesis under different growth and stress conditions and during the sporulation process. Based on this and previous studies, it is now becoming more and more apparent that LTA function is tied to its exact structure, spatial distribution and/or localized synthesis (Schirner *et al*., 2009). To determine the exact function of LTA for bacterial growth and its alterations during different growth conditions warrants further analysis.

## Experimental procedures

### Bacterial strains and growth conditions

All strains used in this study are listed in Table 2. *Escherichia coli* and *S. aureus* strains were grown at 37°C in Luria–Bertani (LB) and tryptic soya broth (TSB) respectively. *B. subtilis* strains were grown in LB, or Difco Antibiotic Medium 3 (PAB medium) at 30°C or 37°C as indicated. When appropriate, the medium was supplemented with antibiotics as indicated in Table 2.

**Table 2:** Bacterial strains used in this study

Strain	Relevant features	Reference
***Escherichia coli* strains**		
XL1 Blue	Cloning strain, TetR – ANG127	Stratagene
DH5α	Cloning strain – ANG397	Hanahan (1983)
Rosetta	Strain used for protein expression – ANG574	Novagen
ANG201	pCN34 in *E. coli*; source for Kan (*aphA*-3) marker; KanR and AmpR	Charpentier *et al*. (2004)
ANG203	pCN49 in *E. coli*; source for Erm (*ermC*) marker; AmpR	Charpentier *et al*. (2004)
ANG204	pCN55 in *E. coli*; source for Spec (*add9*) marker; AmpR	Charpentier *et al*. (2004)
ANG243	pCL55 in XL1 Blue; *S. aureus* single-site integration vector; AmpR	Lee *et al*. (1991)
ANG284	p*itet* in XL1 Blue; *E. coli* / *S. aureus* shuttle vector with tetracycline inducible promoter; AmpR	Gründling and Schneewind (2007b)
ANG503	pCL55-*ltaS* in XL1 Blue; AmpR	This study
ANG506	pCL55-*yqgS* in XL1 Blue; AmpR	This study
ANG508	p*itet*-*ltaS* in XL1 Blue; *ltaS* under tetracycline inducible promoter; AmpR	Gründling and Schneewind (2007a)
ANG509	p*itet*-*yfnI* in XL1 Blue; *yfnI* under tetracycline inducible promoter; AmpR	Gründling and Schneewind (2007a)
ANG510	p*itet*-*yflE* in XL1 Blue; *yflE* under tetracycline inducible promoter; AmpR	Gründling and Schneewind (2007a)
ANG512	p*itet*-*yvgJ* in XL1 Blue; *yvgJ* under tetracycline inducible promoter; AmpR	Gründling and Schneewind (2007a)
ANG1444	pProEX-eYflE in DH5α; plasmid for overexpression of eYflE (eLtaS_BS_) domain; N-terminal His-tag; AmpR	This study
ANG1445	pProEX-eYfnI in DH5α; plasmid for overexpression of eYflnI domain; N-terminal His-tag; AmpR	This study
ANG1446	pProEX-eYvgJ in DH5α; plasmid for overexpression of eYvgJ domain; N-terminal His-tag; AmpR	This study
ANG1447	pProEX-eYqgS in DH5α; plasmid for overexpression of eYqgS domain; N-terminal His-tag; AmpR	This study
ANG1474	pProEX-eYflE in Rosetta strain; strain use for overexpression of eYflE (eLtaS_BS_) protein with N-terminal His tag; AmpR	This study
ANG1475	pProEX-eYfnI in Rosetta strain; strain use for overexpression of eYfnI protein with N-terminal His tag; AmpR	This study
ANG1476	pProEX-eYvgJ in Rosetta strain; strain use for overexpression of eYvgJ protein with N-terminal His tag; AmpR	This study
ANG1477	pProEX-eYqgS in Rosetta strain; strain use for overexpression of eYqgS protein with N-terminal His tag; AmpR	This study
ANG1512	pCN34*itet-ltaS* in XL1 Blue; *ltaS* under tetracycline inducible promoter; KanR, AmpR	This study
ANG1514	pCN34*itet-yfnI* in XL1 Blue; *yfnI* under tetracycline inducible promoter; KanR, AmpR	This study
ANG1615	p*itet*-RB*ltaS-yqgS* in XL1 Blue; *yqgS* with *ltaS* ribosomal binding site under tetracycline inducible promoter; AmpR	This study
ANG1652	pCN34*itet-yqgS* in XL1 Blue; *yqgS* under tetracycline inducible promoter; KanR, AmpR	This study
ANG1656	pCN34*itet-yvgJ* in XL1 Blue; *yvgJ* under tetracycline inducible promoter; KanR, AmpR	This study
ANG1660	pCN34*itet-yflE* in XL1 Blue; *yflE (ltaS_BS_)* under tetracycline inducible promoter; KanR, AmpR	This study
ANG1676	pCN38 in *E. coli*; source for Gram-positive Cam (*cat194*) marker; AmpR	Charpentier *et al*. (2004)
***Staphylococcus aureus* strains**		
RN4220	Transformable laboratory strain	Kreiswirth *et al*. (1983)
ANG499	RN4220 with IPTG-inducible *ltaS* expression; ErmR, IPTG	Gründling and Schneewind (2007a)
ANG514	p*itet*-*ltaS* integrated in strain ANG499; ErmR, CamR, IPTG	Gründling and Schneewind (2007a)
ANG1130	ANG499 with pCN34; ErmR, KanR, IPTG	This study
ANG1571	ANG499 with pCN34*itet-ltaS*; ErmR, KanR, IPTG	This study
ANG1573	ANG499 with pCN34*itet-yfnI*; ErmR, KanR, IPTG	This study
ANG1654	ANG499 with pCN34*itet-yqgS*; ErmR, KanR, IPTG	This study
ANG1658	ANG499 with pCN34*itet-yvgJ*; ErmR, KanR, IPTG	This study
ANG1662	ANG499 with pCN34*itet-yflE*; ErmR, KanR, IPTG	This study
***Bacillus subtilis* strains**		
*B. subtilis*	*Bacillus subtilis* 168 – Transformable lab strain, trpC2 – ANG1691	Burkholder and Giles (1947)
ANG1692	*Bacillus subtilis* 168 *yfnI::Cam* (Δ*yfnI*)	This study
ANG1693	*Bacillus subtilis* 168 *yflE::Kan* (Δ*ltaS_BS_*)	This study
ANG1694	*Bacillus subtilis* 168 *yqgS::Spec* (Δ*yqgS*)	This study
ANG1695	*Bacillus subtilis* 168 *yvgJ::Erm* (Δ*yvgJ*)	This study
ANG1696	*Bacillus subtilis* 168 *yflE::Kan, yqgS::Spec, yvgJ::Erm* (*yfnI only*)	This study
ANG1697	*Bacillus subtilis* 168 *yfnI::Cam, yqgS::Spec, yvgJ::Erm* (*ltaS_BS_ only*)	This study
ANG1698	*Bacillus subtilis* 168 *yfnI::Cam, yflE::Kan, yvgJ::Erm* (*yqgS only*)	This study
ANG1699	*Bacillus subtilis* 168 *yfnI::Cam, yflE::Kan, yqgS::Spec* (*yvgJ only*)	This study
ANG1701	*Bacillus subtilis* 168 *yfnI::Cam, yflE::Kan, yqgS::Spec, yvgJ::Erm* (4xΔ)	This study
ANG1702	*Bacillus subtilis* 168 *yflE::Kan, yfnI::Cam (*express-*yvgJ/yqgS)*	This study
ANG1703	*Bacillus subtilis* 168 *yflE::Kan, yqgS::Spec (*express-*yvgJ/yfnI)*	This study
ANG1704	*Bacillus subtilis* 168 *yfnI::Cam, yqgS::Spec (*express-*yvgJ/yflE)*	This study
L5703	*Bacillus subtilis* with ribitol-Pi wall teichoic acid (ribitol-Pi WTA)	Karamata *et al*. (1987)

Antibiotics were used at the following concentrations: for *E. coli* cultures: ampicillin (AmpR) 100 µg ml^−1^; kanamycin (KanR) 30 µg ml^−1^; tetracycline (TetR) 10 µg ml^−1^; for *S. aureus* cultures: erythromycin (ErmR) 10 µg ml^−1^; chloramphenicol (CamR) 7.5 to 10 µg ml^−1^ and IPTG at 1 mM; for *B. subtilis* cultures: erythromycin 5 µg ml^−1^; chloramphenicol 10 µg ml^−1^; kanamycin 10 µg ml^−1^; spectinomycin (Spec) 100 or 200 µg ml^−1^.

### Strain and plasmid construction

Primers used in this study are listed in Table 3. Plasmids pProEX-eYflE, pProEX-eYfnI, pProEX-eYvgJ and pProEX-eYqgS were constructed for the expression and purification of N-terminally His-tagged versions of the extracellular enzymatic domains of the four *B. subtilis* LtaS orthologues. Respective gene fragments (Fig. S1) were amplified from *B. subtilis* 168 chromosomal DNA using primer pairs 5-BamHI-YflE-Cterm/3-XbaI-YflE with stop, 5-BamHI-YfnI-Cterm/3-XbaI-YfnI with stop, 5-BamHI-YvgJ-Cterm/3-XbaI-YvgJ with stop, 5-BamHI-YqgS-Cterm/3-XbaI-YqgS with stop and cloned BamHI/XbaI into plasmid pProEX-HTb (Invitrogen) that has been cut with the same enzymes. The resulting plasmids were initially transformed into *E. coli* strain DH5α resulting in strains ANG1444 (pProEX-eYflE), ANG1445 (pProEX-eYfnI), ANG1446 (pProEX-eYvgJ) and ANG1447 (pProEX-eYqgS). For protein expression and purification plasmids were introduced into the *E. coli* Rosetta strain yielding strains ANG1474 (pProEX-eYflE), ANG1475 (pProEX-eYfnI), ANG1476 (pProEX-eYvgJ) and ANG1477 (pProEX-eYqgS). Plasmids pCN34*itet-ltaS*, pCN34*itet-yfnI*, pCN34*itet-yflE*, pCN34*itet*-*yvgJ* and pCN34*itet-yqgS* were constructed to study the functions of the corresponding proteins in an *S. aureus ltaS* depletion strain. For the construction of plasmids pCN34*itet-ltaS* and pCN34*itet-yfnI,* respective genes and the p*itet* promoter region were amplified from plasmids p*itet-ltaS* and p*itet-yfnI* using primer pairs 5-KpnI-tet/3-SalI-pCL55 and 5-KpnI-tet/3-P-pCL55 respectively. The resulting PCR products were cut with KpnI/SalI (*ltaS*) or KpnI (*yfnI*) and cloned into pCN34 that has been cut with KpnI/SalI (*ltaS* cloning) or KpnI/SmaI (*yfnI* cloning). For pCN34*itet-yflE*, pCN34*itet*-*yvgJ* and pCN34*itet-yqgS* plasmid construction, the respective genes and the p*itet* promoter were amplified from plasmids p*itet-yflE*, p*itet-yvgJ* and p*itet*-RB*ltaS-yqgS* using primer pair 5-KpnI-tet/3-PstI-pCL55. The resulting PCR products were cut with KpnI and PstI and ligated with pCN34, which has been cut with the same enzymes. Plasmids were subsequently transformed into *E. coli* XL1 Blue resulting in strains ANG1512 (pCN34*itet-ltaS*)*,* ANG1514 (pCN34*itet-yfnI*), ANG1660 (pCN34*itet-yflE*), ANG1656 (pCN34*itet*-*yvgJ*) and ANG1652 (pCN34*itet-yqgS*). These plasmids and the empty pCN34 control vector were then electroporated into the *ltaS*-inducible *S. aureus* strain ANG499, yielding strains ANG1130 (pCN34), ANG1571 (pCN34*itet-ltaS*)*,* ANG1573 (pCN34*itet-yfnI*), ANG1662 (pCN34*itet-yflE*), ANG1658 (pCN34*itet*-*yvgJ*) and ANG1654 (pCN34*itet-yqgS*). Plasmids pCL55-*ltaS* and pCL55-*yqgS*, which were used as DNA templates in PCR reactions for the construction of pCN34*itet-ltaS* and pCN34*itet-yqgS* were constructed as follows: *ltaS* and *yqgS* genes were amplified from *S. aureus* RN4220 and *B. subtilis* 168 chromosomal DNA, respectively, using primer pair 5′-BamHI +P SAV0719/3-KpnI-SAV719 and 5-BamHI-YqgS-with P/3-KpnI-YqgS. Resulting PCR products were cut with BamHI and KpnI and ligated with plasmid pCL55, which had been cut with the same enzymes. Plasmids were subsequently transformed into *E. coli* strain XL1 Blue resulting in strains ANG503 (pCL55-*ltaS*) and ANG506 (pCL55-*yqgS*). Plasmid p*itet*-RB*ltaS-yqgS*, which was used to amplify *yqgS* for the construction of pCN34*itet-yqgS*, contains the *yqgS* gene proceded by the *S. aureus ltaS* RBS under tetracycline inducible promoter control. This plasmid was obtained by amplifying the *ltaS* promoter and RBS from plasmid pCL55-*ltaS* using primer pair 5′-BamHI +P SAV0719/R-YqgS-PLtaS and the *yqgS* coding sequence from plasmid pCL55-*yqgS* using primer pair N-F-PltaS-YqgS/3-BglII-YqgS. The resulting PCR products were fused by SOE (Splice Overlap Extension) PCR (Horton *et al*., 1989) using primer pair 5′AvrII-PltaS-yqgS/3-BglII-YqgS. The final PCR product was cut with AvrII/BglII and *yqgS* with the *ltaS* RBS was placed under tetracycline inducible promoter control by ligating the cut PCR product with the AvrII/BglII cut plasmid p*itet*. The resulting plasmid p*itet*-RB*ltaS-yqgS* was transformed into *E. coli* strain XL1 Blue, yielding strain ANG1615. Sequences of all inserts were verified by fluorescence automated sequencing at the MRC Clinical Science Center Sequencing Facility at Imperial College London.

**Table 3:** Primers used in this study

Number	Name	Sequence
ANG086	5′-BamHI +P SAV0719	CGGGATCCGGAATAGAATATAGAATGCAATTAGAAATG
ANG159	5-KpnI-tet	GGGGTACCTTGGTTACCGTGAAGTTACCATCACGG
ANG317	3-KpnI-SAV719	GGGGTACCCCGAGTTCGTGTTTAAATATTATTTTTTAG
ANG322	5-AvrII-YfnI-33bp	CCGCCTAGGGAACTTAAAGTGTTTAAGAAAGTAGAGGTTGCC
ANG323	3-BglII-YfnI	GAAGATCTGCAATGCGCCCGCTCAAGGCTCTTTTTCATCTTA
ANG326	5-AvrII-YflE-32bp	CCGCCTAGGGCTCGAACTGGATCGGAAAAAAGGAGTGTAACA
ANG327	3-BglII-YflE	GAAGATCTAAAGCGGAGAGGGCAACCTCTCCGCTTTTTCTTA
ANG328	5-BamHI-YqgS- with P	CGGGATCCGTCGAGAAAACATTCCGCAAATGCGCGTTTCCG
ANG329	3-KpnI-YqgS	GGGGTACCCCGCTCACTTCGATGCGGGAGACATTGTGATTA
ANG330	5-AvrII-YqgS-34bp	CCGCCTAGGCTGATTTTTTTGAGCGTGCTGCATAGGAGGTTG
ANG331	3-BglII-YqgS	GAAGATCTCCGCTCACTTCGATGCGGGAGACATTGTGATTA
ANG334	5-AvrII-YvgJ-33bp	CCGCCTAGGCAGATGATCAAGAAAACGTGAGGAGTCATATTG
ANG337	3-BeglII-YvgJ (2)	GAAGATCTGGACTACAAGGCGAATCTGTCTCATTTAAA
ANG579	5-BamHI-YflE-Cterm	CGGGATCCGATTCCAGCGACGTAACGGAAGTAG
ANG580	3-XbaI-YflE with stop	CGTCTAGAGGGCAACCTCTCCGCTTTTTCTTATTTATC
ANG581	5-BamHI-YfnI-Cterm	CGGGATCCAGCAGCGATGATTTAACAAGTGTCGAG
ANG582	3-XbaI-YfnI with stop	CGTCTAGAGCGCCCGCTCAAGGCTCTTTTTCATCTTA
ANG583	5-BamHI-YvgJ-Cterm	CGGGATCCGATGAAGACAGTATAACTGCCATTAAAAAC
ANG584	3-XbaI-YvgJ with stop	CGTCTAGACCCTCTTGATAGAGGGATTTTTTCA
ANG585	5-BamHI-YqgS-Cterm	CGGGATCCGACAGCAACAGCCTGACGGAGATTG
ANG586	3-XbaI-YqgS with stop	CGTCTAGACTTCGATGCGGGAGACATTGTGATTA
ANG775	R-YqgS-PLtas	CGTTTTTCGCATGATTCTTTCCCCCGTTATTTAGATAATAAATC
ANG796	N-F-PltaS-YqgS	GGGGAAAGAATCATGCGAAAAACGTTTTTTTCGAAGATTTC
ANG800	3-SalI-pCL55	ACGCGTCGACCACGTTTCCATTTATCTGTATACGGATC
ANG821	3-P-pCL55	P-CACGTTTCCATTTATCTGTATACGGATC
ANG826	5′AvrII-PltaS-yqgS	CCGCCTAGGCTAAATAACGGGGGAAAGAATCATG
ANG877	3-PstI-pCL55	CGGCTGCAGCACGTTTCCATTTATCTGTATACGGATC
ANG1070	5-1kb-YfnI	CTTCCGAAAGACCCTGAAACGC
ANG1071	3-ApaI-YfnI	CCGGGGCCCGGCAACCTCTACTTTCTTAAACAC
ANG1072	5-XhoI-YfnI	CCGCTCGAGCTATCATTACGGCAAGGAGAAAGAAATC
ANG1073	3-1kb-YfnI	CCCATCTTTGGCAAGGTTCTTCAGC
ANG1076	5-1kb-YflE	GATTGTCTGTTTGAAAATGTATAAAGG
ANG1077	3-ApaI-YflE	CCGGGGCCCTGTTACACTCCTTTTTTCCGATCCAG
ANG1078	5-XhoI-YflE	CCGCTCGAGCTTCCGAAACGTCAAAGGATAACGAAG
ANG1079	3-1kb-YflE	CAACTCGTTTGGAGAGTGGATGCTC
ANG1082	5-1kb-YqgS	GCAAGCTGGATGAGCTGCAAAAACC
ANG1083	3-ApaI-YqgS	CCGGGGCCCCATTCAACCTCCTATGCAGCACGCTC
ANG1084	5-XhoI-YqgS	CCGCTCGAGCGGAAAGAAAAACAAATGCTTGATC
ANG1085	3-1kb-YqgS	CGGCTCCGTAAGCGGAGAGATTCCC
ANG1088	5-1kb-YvgJ	TAGAGCCAAGCCCTATCCGCATGTGG
ANG1089	3-ApaI-YvgJ	CCGGGGCCCCAATATGACTCCTCACGTTTTCTTG
ANG1090	5-XhoI-YvgJ	CCGCTCGAGGTGACCTGCTCAGGTTTTCCGAATG
ANG1091	3-1kb-YvgJ	CTTACCGTGTCGGAAGGCAGATGCG

Relevant restriction sites in primer sequences are underlined.

*Bacillus subtilis* single, double, triple and quadruple *yfnI*, *yflE (ltaS_BS_)*, *yqgS* and *yvgJ* mutants were constructed by replacing each gene with an antibiotic resistance marker (*yfnI::Cam*, *yflE::Kan*, *yqgS::Spec*, *yvgJ::Erm*)*.* The initial single mutants were obtained by transforming PCR products composed of the antibiotic resistance cassette flanked by ∼900 bp upstream and downstream DNA fragments of the respective gene. Subsequent mutants were produced using chromosomal DNA of appropriate single mutants in transformation reactions. Initial PCR products were obtained as follows: primer pairs 5-1kb-YfnI/3-ApaI-YfnI, 5-1kb-YflE/3-ApaI-YflE, 5-1kb-YqgS/3-ApaI-YqgS and 5-1kb-YvgJ/3-ApaI-YvgJ were used to amplify ∼900 bp fragments upstream of each gene and the resulting PCR products were cut with ApaI. Primer pairs 5-XhoI-YfnI/ 3-1kb-YfnI, 5-XhoI-YflE/ 3-1kb-YflE, 5-XhoI-YqgS/3-1kb-YqgS and 5-XhoI-YvgJ/3-1kb-YvgJ were used to amplify ∼900 bp fragments downstream of the respective genes and the resulting PCR products were cut with the restriction enzyme XhoI. Antibiotic resistance cassettes were excised with ApaI and XhoI from plasmids pCN38 (Cam), pCN34 (Kan), pCN54 (Spec) and pCN49 (Erm) and appropriate upstream DNA fragments – resistance marker – downstream DNA fragments were ligated overnight. An aliquot of these ligation reactions was used for a final PCR amplification step together with the outside primers 5-1kb-YfnI/3-1kb-YfnI, 5-1kb-YflE/3-1kb-YflE, 5-1kb-YqgS/3-1kb-YqgS and 5-1kb-YvgJ/3-1kb-YvgJ. *B. subtilis* transformations were performed using the two-step transformation procedure as described previously (Cutting & Vander Horn, 1988) and transformants were selected on LB agar plates containing the appropriate antibiotic. All mutations were verified by PCR using primer pairs 5-AvrII-YfnI-33bp/3-BglII-YfnI, 5-AvrII-YflE-32bp/3-BglII-YflE, 5-AvrII-YqgS-34bp/3-BglII-YqgS and 5-AvrII-YvgJ-33bp/3-BglII-YvgJ (2) and chromosomal DNA, which was prepared using the FastDNA kit (MP-Biomedicals). Replacement of *yfnI, yflE, yqgS* and *yvgJ* genes with the resistance marker resulted in a PCR product of reduced size.

### Protein purification

Protein expression, Ni-affinity and size exclusion protein purification were undertaken as described previously with the modification that protein expression was induced at an OD_600_ of 0.4 by the addition of 0.5 mM isopropyl β-d-1-thiogalactopyranoside (IPTG) (Lu *et al*., 2009). Fractions containing the purified protein were pooled and concentrated using Amicon centricons with a 10 kDa cut-off. The protein concentration was measured using the BCA kit from Pierce and the purity of the protein was estimated by separating 10 µg purified protein on 10% SDS-PAGE gels and Coomassie staining.

### *In vitro*enzyme assay for LtaS-type enzymes

The enzymatic activity of purified proteins was measured by following the hydrolysis of NBD-labelled PG (NBD-PG) using a method described previously (Karatsa-Dodgson *et al*., 2010). Briefly, 1.8 ml of 10 mM sodium succinate buffer, pH 6.0, ionic strength (µ) = 50 mM (adjusted with NaCl) was added to 25 µg of TLC-purified NBD-PG lipid (Avanti; order number 810163). Lipids were brought into suspension by sonication for 45 s at 11 amplitude microns using a Soniprep Sanyo sonicator. Next, MnCl_2_ or other divalent cations were added from a 1 M stock solution to give a final concentration of 10 mM, the samples were vortexed and 303 µl (∼4.166 ng lipid) aliquots transferred into test tubes. Reactions were initiated by the addition of 1.52 µM purified protein and assay mixtures were then incubated for 3 h in a 37°C water bath. Next, reactions were stopped and lipids extracted by the addition of CHCl_3_/MeOH to give a final ratio of assay volume : CHCl_3_ : MeOH of 0.9:1:1. Tubes were vortexed vigorously, centrifuged for 5 min at 17 000 *g* and fractions of the bottom chloroform phase transferred to a new tube and dried under a stream of nitrogen. Dried lipids were then suspended in 10 µl chloroform, spotted onto pre-run Å60 silica gel plates (Macherey-Nagel) and separated using a chloroform: methanol: H_2_O (65:25:4) solvent system. Plates were dried and subsequently scanned using a Fujifilm FLA-5000 imager equipped with a 473 nm excitation laser and a FITC emission filter. Where indicated, fluorescent signals of lipid reaction products were quantified using the AIDA software (Raytest Isotopenmessgeräte GmbH). The phospholipase PC-PLC (PLC) from *B. cereus* (Sigma EC 3.1.4.3) was used as a positive control enzyme and assays were set up as described previously (Karatsa-Dodgson *et al*., 2010). To gain insight into enzyme kinetics, a time-course experiment was performed by removing and analysing samples at the indicated time points. Reaction products were quantified using a fluorescence plate reader and the AIDA software and per cent hydrolysis of input lipid calculated based on the signal obtained for the PLC control reaction, which proceeds to near completion. Reactions were set up with triplicate samples and average values and standard deviations were plotted. Experiments were performed three times and a representative result is shown. Maximal enzyme reaction rates were determined from the slope of the linear fit through the first three data points and average values with standard deviations from the three independent experiments calculated. To determine the enzyme specificity, the fluorescently labelled lipids 16:0-6:0 NBD-PC (Avanti 810130), 16:0-6:0 NBD-PE (Avanti 810153) and 16:0-6:0 NBD-PS (Avanti 810192) were purified on TLC plates and enzyme reactions set up as described above. At least three independent experiments were performed for all presented data and a representative graph is shown.

### *S. aureus*growth curves, LTA and protein detection by Western blot

For growth curves, *ltaS* inducible *S. aureus* strains ANG1130 (pCN34), ANG1571 (pCN34*itet-ltaS*), ANG1573 (pCN34*itet-yfnI*), ANG1662 (pCN34*itet-yflE*), ANG1658 (pCN34*itet*-*yvgJ*) and ANG1654 (pCN34*itet-yqgS*) were grown overnight at 37°C in 4 ml TSB medium containing 1 mM IPTG and appropriate antibiotics. The following day, bacteria from 1 ml culture were washed three times with 1 ml TSB by centrifugation and resuspension. Next, 5 ml TSB containing 300 ng ml^−1^ anhydrotetracycline (Atet) and appropriate antibiotics were inoculated with 50 µl washed bacterial suspensions (1:100 dilution) and cultures incubated at 37°C with shaking. At time 0 and 2 h intervals, culture aliquots were removed and OD_600_ values determined. At 4 h all cultures and at 8 h indicated cultures were diluted 1:100 into 5 ml fresh TSB containing 300 ng ml^−1^ Atet and appropriate antibiotics to maintain bacteria in the logarithmic growth phase. In addition, 4 h after the initial dilution, 1 ml aliquots were withdrawn for sample preparation for Western blot analysis of cell-associated LTA. Samples for Western blot analysis were prepared as described previously (Gründling and Schneewind, 2007a). Samples were normalized for OD_600_ readings of the bacterial cultures; that is, samples from a culture with an OD_600_ of 1 were suspended in 15 µl sample buffer. Samples were boiled for 20 min, centrifuged at 17 000 *g* for 5 min and 10 µl aliquots separated on a 15% SDS-PAGE gel. For Western blot analysis, the polyglycerolphospate-specific LTA antibody (Clone 55 from Hycult biotechnology) and HRP-conjugated anti-mouse IgG (Cell Signaling Technologies, USA) were used at 1:5000 and 1:10 000 dilutions respectively. Antibody incubations were performed in Tris-buffered saline pH 7.6, 0.1% Tween 20 (TBST) buffer containing 5% milk and 10 µg ml^−1^ human IgG and blots were developed by enhanced chemiluminesce. For LTA detection in *B. subtilis*, wild-type and mutant strains were grown for 20–22 h at 30°C in 5 ml PAB medium with shaking. Samples for LTA analysis were prepared from whole cells as follows: Bacteria from 3–4 ml culture were collected by centrifugation for 30 min at 17 000 *g*. Bacterial pellets were suspended in 2× protein sample buffer normalized for OD_600_ readings; that is 100 µl 2× sample buffer was used per ml culture of OD_600_ = 3. Samples were boiled for 45 min, centrifuged for 5 min and 10 µl analysed on a 15% SDS-PAGE gel. The humanized monoclonal LTA antibody (Biosynexus Incorporated; Gaithersburg, MD, USA) and the HRP-conjugated polyclonal rabbit anti-human IgA, IgG, IgM, Kappa, Lambda antibody (DakoCytomation) were used at 1:10 000 dilutions for LTA detection. Antibody incubations were performed in TBST buffer containing 3% BSA. All experiments were performed at least three times and a representative graph is shown.

### *S. aureus*lipid extraction and glycolipid analysis by TLC

For *S. aureus* lipid extraction and glycolipid analysis, strains ANG1130 (pCN34), ANG1571 (pCN34*itet-ltaS*), ANG1573 (pCN34*itet-yfnI*), ANG1662 (pCN34*itet-yflE*), ANG1658 (pCN34*itet*-*yvgJ*) and ANG1654 (pCN34*itet-yqgS*) were grown overnight at 37°C in 10 ml TSB supplemented with 1 mM IPTG and appropriate antibiotics. The next day, cells were collected by centrifugation for 10 min at 1300 *g* and bacterial pellets washed three times with 10 ml TSB. After the final centrifugation step, washed cultures were suspended in 10 ml TSB and diluted 1:100 into 200–800 ml fresh TSB medium supplemented with 300 ng ml^−1^ Atet and appropriate antibiotics. Cultures were incubated at 37°C with shaking for 5 h. Next, bacteria were collected by centrifugation for 10 min at 8000 *g*, washed once with 5 ml of ice-cold 0.1 M sodium citrate buffer pH 4.7 and suspended in 3 ml of the same buffer. Cultures were subsequently dispensed into three 2 ml Fast Prep tubes containing 0.5 ml of 0.1 mm glass beads. Bacteria were lysed and lipids extracted using a modified Bligh-Dyer method as described previously (Kates, 1972; Gründling and Schneewind, 2007a). Lipids were dried under a stream of nitrogen and suspended in chloroform to a final concentration of 50 mg ml^−1^. Ten microlitres (500 µg lipids) was spotted onto pre-run Å60 silica gel plates (Macherey-Nagel) and lipids separated using a chloroform : methanol : H_2_O (65:25:4) solvent system. Glycolipids were visualized by spraying plates with 0.5–1.5% α-naphthol in 50% methanol and then with 95% H_2_SO_4_ (Gründling and Schneewind, 2007a; Kates, 1972). Experiments were performed three times and a representative result is shown.

### Lipid analysis by MALDI mass spectrometry

The MALDI mass spectrometry analysis was essentially performed as described previously (Webb *et al*., 2009). Briefly, total membrane lipids were isolated from *S. aureus* strains ANG1662 (expressing LtaS_BS_) or ANG1658 (expressing YvgJ) and 5 × 0.5 mg lipids (2.5 mg total) were spotted and separated on a TLC plate using the same solvent system as described above. Areas containing glycolipids were determined by developing one lane run in parallel with α-naphthol/H_2_SO_4_. Appropriate silica gel areas were scraped off and lipids extracted as described previously (Gründling and Schneewind, 2007a). Dried lipids were suspended in 10 µl of 0.5 M 2,5-dihydroxybenzoic acid (DHB) MALDI matrix dissolved in 1:1 methanol: chloroform and 1 µl was spotted directly onto MALDI plates or diluted 1:10 using 0.5 M DHB matrix and 1 µl spotted. Spotted MALDI plates were run on a MALDI micro MX machine (Waters, UK) and spectra recorded in the reflector positive ion mode. As additional calibration standard, 25–50 pmol of bradykinin peptide standard (Sigma) with an absolute mass of 7 573 997 (M + H^+^) was spotted in α-cyano-4-hydroxycinnamic acid (CHCA) matrix, which was suspended at 10 mg ml^−1^ in 70% acetonitrile 0.1% TFA. Representative spectra from three independent experiments are shown.

### Microscopy

Wild-type and mutant *B. subtilis* strains (ANG1691 – ANG1699 and ANG 1701) were grown overnight at 30°C in 5 ml PAB medium. Overnight cultures were processed for microscopy analysis as described below or were back-diluted 1/50 into fresh PAB medium and grown for 3 h at 37°C with shaking. Subsequently, an equivalent of a 1 ml culture aliquot with an OD_600_ = 0.5 was removed and bacteria washed three times by centrifugation and suspension in 1 ml PBS pH 7.4 buffer. A small aliquot of washed culture was mounted on poly-lysine coated microscope slides and examined under a Zeiss Axiovert 200 wide field microscope using a 100× objective and images were taken and analysed using the Improvision Volocity software. Two independent experiments were performed and representative images are shown for each strain.

### LTA purification

Lipoteichoic acid was purified from *S. aureus* strains using a previously described 1-butanol extraction method (Morath *et al*., 2001; Gründling and Schneewind, 2007a). Briefly, strains ANG514 (expressing LtaS) and ANG515 (expressing YfnI) were grown overnight at 37°C with shaking in 150 ml TSB supplemented with 1 mM IPTG and appropriate antibiotics. The following day, cultures were centrifuged for 10 min at 1300 *g* and washed three times with 150 ml TSB. Washed cultures were diluted 1:100 into 6 l (strain ANG514) or 12 l (strain ANG515) fresh TSB containing 300 ng ml^−1^ Atet and appropriate antibiotics and incubated at 37°C with shaking for 4–5 h. LTA extraction and purification by hydrophobic interaction chromatography was performed as described previously using a 24 × 1.6 cm octylsepharose column (Gründling and Schneewind, 2007a). LTA-containing fractions were identified by Western blot, pooled and dialysed in the cold 7–11 times against 4 l ddH_2_O and subsequently lyophilized.

### LTA structure analysis by NMR and biochemical assays

For NMR analysis, 1 mg purified LTA was suspended in 500 µl D_2_O of 99.96% purity and lyophilized. This procedure was repeated once and the lyophilized LTA sample was then suspended in 500 µl D_2_O of 99.99% purity and 1d ^1^H NMR spectra recorded at 600 MHz (^1^H) and 300 K on a Bruker AvanceIII spectrometer equipped with a TCI cryoprobe. To ensure accurate integrals of species with potentially differing ^1^H T_1_ values, spectra were recorded with a total recycle time of 6 s and a ^1^H flip angle of *c.* 60°. The length of the glycerolphosphate chain as well as the percentage of d-alanine substitution was calculated from the ^1^H NMR integrals of the appropriate LTA-specific peaks. The LTA length calculation was based on a C15:C18 fatty acid composition (59 non-exchangeable protons from CH_2_/CH_3_ groups), which is the most abundant lipid anchor present in *S. aureus* LTA (Fischer, 1994). NMR analysis was performed on LTA samples isolated from three independent cultures.
