# Supplementary material for: Enzymatic activities and functional interdependencies of Bacillus subtilis lipoteichoic acid synthesis enzymes
Source: Mol Microbiol. 2011 Feb;79(3):566–83. doi: 10.1111/j.1365-2958.2010.07472.x (PMC3089822; doi:10.1111/j.1365-2958.2010.07472.x)
Supplement: Supplementary file 1 [file mmi0079-0566-SD1.pdf]

```
CLUSTAL 2.0.12 multiple sequence alignment

LtaS            MSSQKKKISLFAFFLLTVITITLKTYFSYYVDFSLGVKGLVQNLILLMNPYSLVALVLSVFLFFKGKKAFWFMFIGGFLLTFLLYANVVYFRFFSDFLTFSTLNQVG-NVESMGGAVSAS 119
YfnI            --MKKLFSYKLSFFVLAVILFWAKTYLSYKTEFNLGVKGTTQEILLIFNPFSSAVFFLGLALLAKGRKSAIIMLIIDFLMTFVLYANILFYRFFDDFLTFPNIKQSG-NVGNMGDGIFSI 117
YflE            -MKTFIKERGLAFFLIAVVLLWIKTYVGYVLNFNLGIDNTIQKILLFVNPLSSSLFFLGFGLLFKKKLQQTAIIVIHFLMSFLLYANIVYYRFFNDFITIPVIMQAKTNGGQLGDSAFSL 119
YqgS            --MRKTFFSKISFMLIAILLMWLKTYAVYKTSFHIKIDNLTQEFILFINPLSFLLLIFGLSLFLKGKNRNRYIIAMSCLVTFVLLANMVFYRFYNDFLTIPVLFQTS-NMGDLGSSIGTL 117
YvgJ            --MKGTFFHNQRFLCFSILFMWIKTYVIYKLGFDLQIDTLLEELMLLVNPLSFILPLFGIGLFLKENKQRAFLLIANLVLTVILISNTIFYGFYIDFITIPVLFQAS-NMSDMGSSVKEL 117
                             *: :::: :  ***  *   * : :.   ::::*:.** *    .:.. *: * .     ::    :::.:* :* ::: *: **:*:. : *   *  .:*..

LtaS            FKWYDFVYFIDTLVYLFILIFKTKWLDTKAFSKKFVPVVMAASVALFFLNLAFAETDRPELLTRTFDHKYLVKYLGPYNFTVYDGVKTIENNQQKALASEDDLTKVLNYTKQRQTEPNPE 239
YfnI            MAGHDIFYFLDIIILIAVLIWRPELKEYK-MKKRFASLVILSGIALFFINLHYAEKDRPQLLTRTFDRNYIVKYLGLYNYTIYDGVQTAQTETQRAYASSDDLTSVENYTTSHYAKPNAE 236
YflE            MRPTDAFYFIDTIILIIILAIKVNKPAETS--SKKSFRIIFASSILVFLINLAVAESDRPELLTRSFDRNYLVKYLGTYNFTIYDAVQNIKSNSQRALADSSDVTEVENYMKANYDVPNNV 237
YqgS            LEPTDLLLAVDIAVLIWLHIRQKAFQSDIPSTKNERAAYFLFVASVYFFNLGLSEAERPQLLTRSFDREMLVKNISLFNFHIYDGVLQSKQSAQRALADSNSLTEIENYVTANAKDANKR 237
YvgJ            FHPLFIALFVDLVFLLLFARKTKHPQTKAAPHTIKR--YYAASCGMLLCTLALAEVQQPKLLAHSFDREMLVKSIGLFQFHIYDTISQTVNISAKAFADEDSITAIKNYTEADYSKPDQS 235
                :        :*  . : .              .           : : .*  :* ::*:**:::**:: :** :. ::: :** :          :* *....:* : **       .:

LtaS            YYGVAKKKNIIKIHLESFQTFLINKKVNGKEVTPFLNKLSSGKEQFTYFPNFFHQTGQGKTSDSEFTMDNSLYGLPQGSAFSLKGDNTYQSLPAILDQKQGYKSDVMHGDYKTFWNRDQV 359
YfnI            YFGSAKGKNIIKIHLESFQSFLIDYKLNGEEVTPFLNKLAHGGEDVTYFDNFFHQTGQGKTSDAELTMDNSIFGLPEGSAFVTKGENTYQSLPAILDQKEGYTSAVLHGDYKSFWNRDQI 356
YflE            YFGKAEGKNVIYVSLESLQSFIIDYKIDGKEVTPFLNKLAHDNETF-YFDNFFHQTGQGKTSDAEFMMENSLYPLAQGSVFVNKAQNTLQSVPAILKSKN-YTSATFHGNTQTFWNRNEM 355
YqgS            LFGAAKGRNVILVSLESTQSFVINEKLNGEEITPFLNDFIK---QSYNFNNVYHQTGQGKTSDSEFIVDNSLYPLGRGAVFFTNAGNQYMAAPEILKNSG-YYSAVLHANNKSFWNRDLM 353
YvgJ            KFGLAKGRNVIFVTLESTQSFVLNEKVNGKEITPFMNDLIK---KSYSFDHFYQQTEQGKTSDSEFIVANSLYPSLSGAVFFTKSDHQFHTMYKSLKQHD-YYSAVFHANHKTFWNRDVM 351
                 :* *: :*:* : *** *:*::: *::*:*:***:*.:           * :.::** *******:*: : **::     *:.*  :. :   :    *..   * * .:*.: ::****: :

LtaS            YKHFGIDKFYDATYYDMSDKNVVNLGLKDKIFFKDSANYQAKMKSPFYSHLITLTNHYPFTLDEKDATIEKSNTGDATVDGYIQTARYLDEALEEYINDLKKKGLYDNSVIMIYGDHYGI 479
YfnI            YKHIGYDKFFDASTYDMSDENVINMGLKDKPFFTESIPKLESLKQPFYAHLITLTNHYPFNLDEKDASLKKATTGDNTVDSYFQTARYLDEALEQFFKELKEAGLYDNSVIMIYGDHNGI 476
YflE            YKAEGIDKFFDSAYYDMNEENTKNYGMKDKPFFKESMPLLESLPQPFYTKFITLSNHFPFGMDEGDTDFPAGDFGDSVVDNYFQSAHYLDQSIEQFFNDLKKDGLYDKSIIVMYGDHYGI 475
YqgS            YDSFGYDSFFDINSYDVTDENSVGWGLKDKEFFEQSSELMKNLPQPFYSRLITLTNHFPFDLDEEDQLIDEYDSNSQTLNKYFPTVRYQDEALKRFIEKLKEDGLYDNSVIVLYGDHYGI 473
YvgJ            YDTFGIDRFFDVDDFHVTPGTSTSWGLKDKEFLEQSAKKLKSLPQPFYSSFITLTNHFPFEIDEKDQLIDEFDSSSDLLNRYVTTVRYEDEALKHFIKKLKDEGLYENSMIVFMGDHYGI 471
                *.  * * *:*   :.:.  .  . *:*** *: :*     .: .***: :***:**:** :** *  :      ..  :: *. :.:* *::::.:::.**. ***::*:*:: *** **

LtaS            SENHNNAMEKLLG-EKITPAKFTDLNRTGFWIKIPGKSG---GINNEYAGQVDVMPTILHLAGIDTKNYLMFGTDLFSKGHNQVVPFRNGDFITKDYKYVNGKIYSNKNNELITTQPADF 595
YfnI            SENHNRAMKEILG-KEITDYQNAQNQRVPLMIRVPGKKG---GVNHTYGGEIDVMPTLLHLEGIDSQKYINFGTDLFSKDHDDTVAFRNGDFVTPKYTSVDNIIYDTKTGEKLKAN-EET 591
YflE            SENHNKAMAKVLGKDEITDYDNAQLQRVPLFIHAAGVKG---EKVHKYAGDVDVAPTILHLLGVDTKDYLMSGSDILSKEHREVIPFRNGDFISPKYTKISGKYYDTKTGKELDES-EVD 591
YqgS            SENHNEAMGQFLGK-EITPFEEVQLQKVPLVIHIPGITDKKPQTIETVGGQIDIRPTLMNLLGIDTKDQIQFGNDLLSDEKLDFTVLRDGSFITDQVVYTDGACYDKETGKPLEET-KQC 591
YvgJ            SEAHNEAMAEFLGKDEITPYDNVQLQRVPFIIHIPGITDQQPETIPDAGGQVDVRPTLMHLLGVETKGDIQFGNDLLSGDRTPFAVLRNGSFITNDYVYTKNTCYSQKTGEVLEDQ-DAC 590
                ** **.** :.**  :**  . .: ::. : *: .* ..        .*::*: **:::* *::::  :  *.*::*  :      :*:*.*:: .    ..  *. :..: :

LtaS            EKNKKQVEKDLEMSDNVLNGDLFRFYKNPDFKKVNPSKYKYETGPKANSKK------- 646
YfnI            KNLKTRVNQQLSLSDSVLYKDLLRFHKLNDFKAVDPSDYHYGKEKEIK---------- 639
YflE            KSEDSLVKKELEMSDKIINGDLLRFYEPKGFKKVNPSDYDYTKHDEDSSETSKDNEDK 649
YqgS            EAFADKAKQELSLSDEIIYGDLLRFYDQKRLDNSSKRKEKQMLDQAS----------- 638
YvgJ            LPYKEKANEELSLSDKILNGDLLRFSE------------------------------- 617
                       .:::*.:**.::  **:** .
```

Supplemental Fig. S1. ClustalW alignment of *S. aureus* LtaS and *B. subtilis* 168 $LtaS_{BS}$ (YflE), YqgS and YvgJ proteins. The active site threonine residue is highlighted by a red box and the region highlighted in blue corresponds to the extracellular enzymatic domain, which was cloned, expressed and purified as N-terminal His-tagged fragment from *E. coli* extracts. The extracellular enzymatic domains are preceded by an AXA motif (highlighted in grey), which is proposed to be a signal peptidase cleavage site.

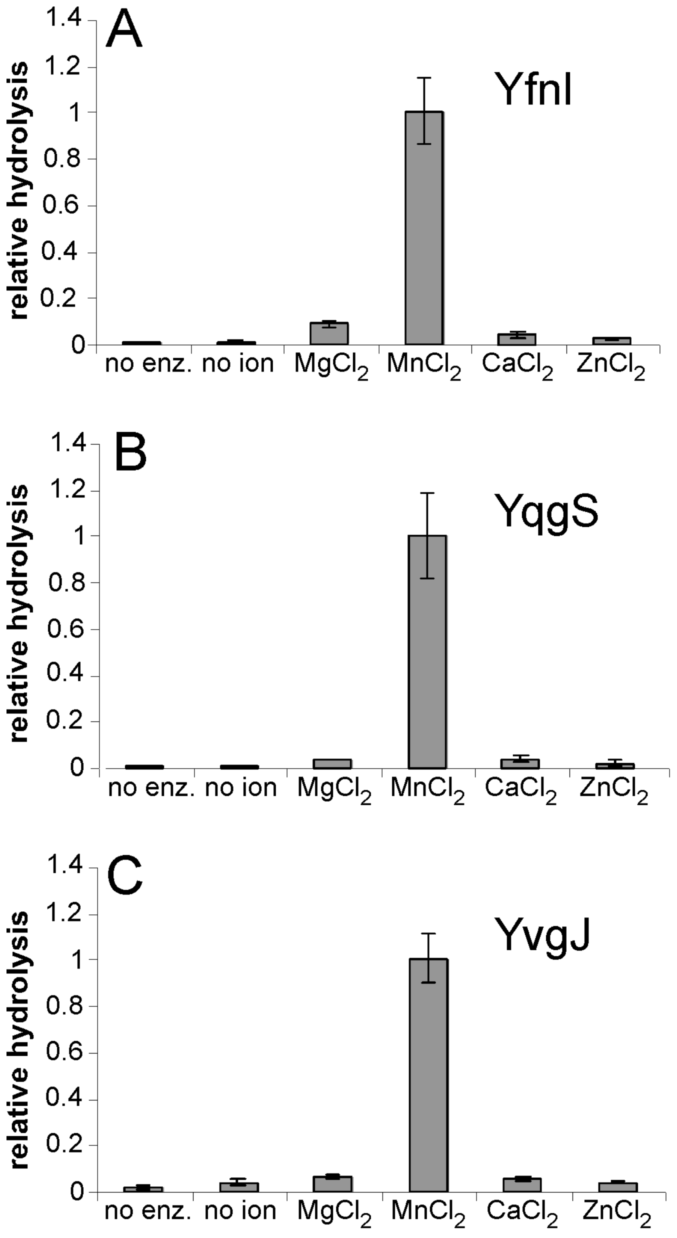

Supplemental Fig. S2. Recombinant *B. subtilis* enzymes YfnI, YqgS and YvgJ require Mn^{2+} for activity. Enzyme assays were set up in 10 mM sodium succinate pH 6, $\mu = 50$ mM buffer in the presence of 10 mM $MgCl_2$, $MnCl_2$, $CaCl_2$ or $ZnCl_2$ and reactions were initiated by the addition of (A) eYfnI, (B) eYqgS or (C) eYvgJ. As controls, reactions were set up without enzyme or without metal ion added. Samples were incubated for 3 h at 37°C and lipids were subsequently extracted and separated on TLC plates. The reaction product was quantified using a fluorescence imager and the AIDA software. The average fluorescence value for the reaction product of samples containing $MnCl_2$ was arbitrarily set to one in all graphs and other values adjusted accordingly. At least three independent experiments were performed and a representative graph is shown.

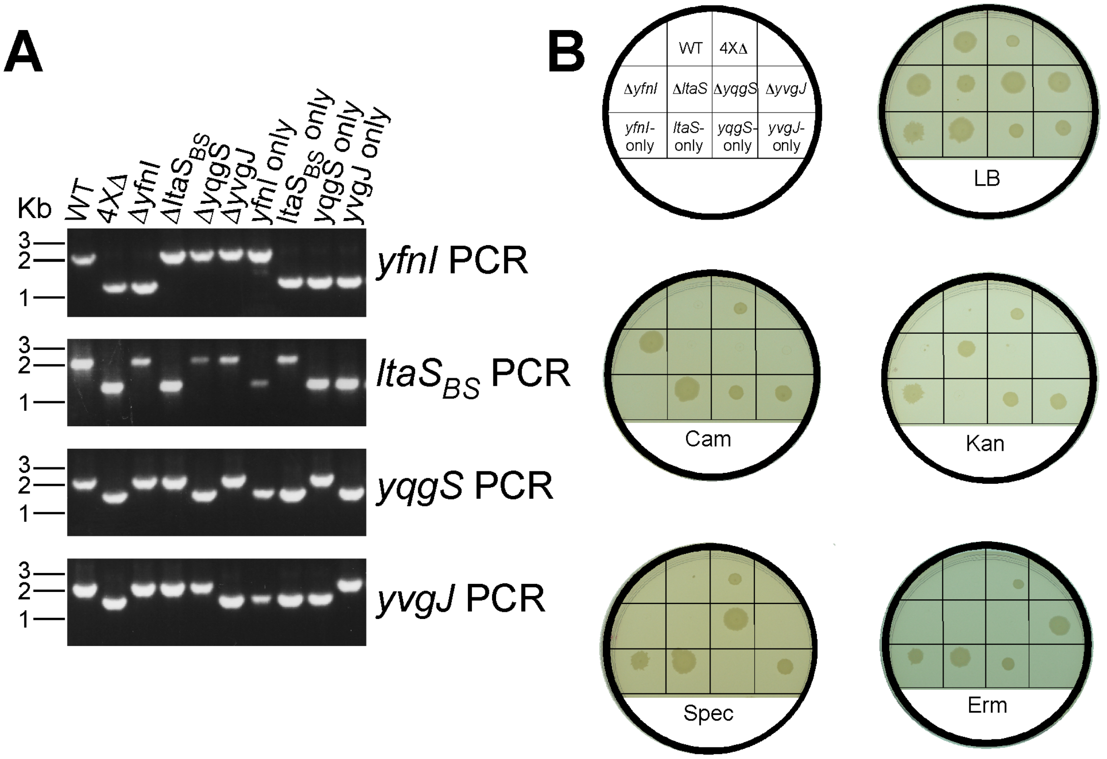

Supplemental Fig. S3. Confirmation of *B. subtilis* single, triple and the quadruple mutant by PCR and antibiotic resistance test. (A) Mutant analysis by PCR. Chromosomal DNA was isolated from wild type and mutant *B. subtilis* strains and used in PCR reactions with *yfnI*, $ltaS_{BS}$ *(yflE)*, *yqgS* and *yvgJ* gene specific primers pairs listed in Table 3. Resultant PCR products were analyzed on a 0.8% agarose gel and replacement of respective genes with an antibiotic marker resulted in a decrease in PCR product size. Chromosomal DNA used is indicated above each lane and the gene specific primer set used is shown on the right of each panel. Sizes of DNA marker fragments run in parallel are shown on the left. (B) Antibiotic resistance profile of wild type and mutant *B. subtilis* strains. Four µl of the indicated *B. subtilis* overnight cultures (top left) were spotted on LB plates, or LB plates containing Cam (10 µg/ml), Kan (10 µg/ml), Spec (100 µg/ml) or Erm (5 µg/ml) and pictures were taken after overnight growth at 37°C. PCR sizes and antibiotic resistance profile correlate with the expected gene deletions in each strain.

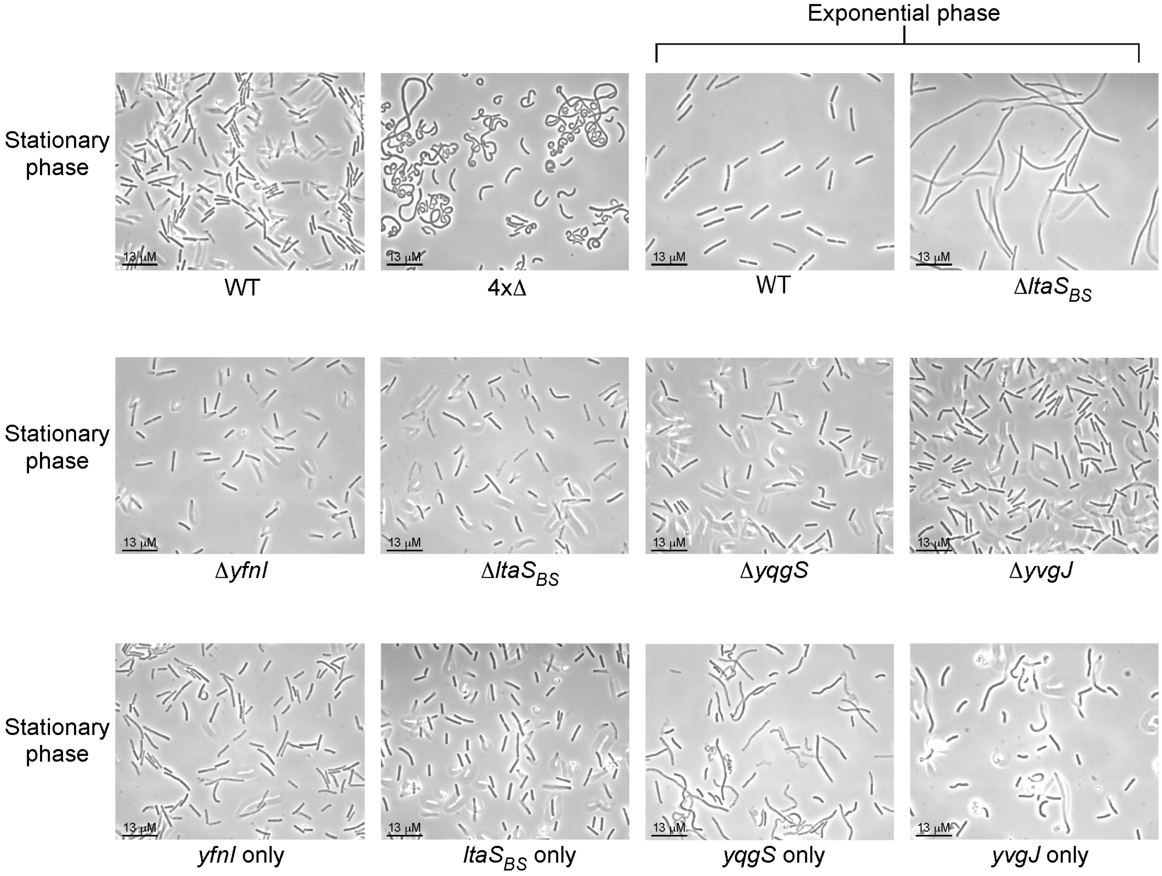

Supplemental Fig. S4. Microscopy analysis of wild type and mutant *B. subtilis* strains. Wild type and mutant *B. subtilis* strains were grown over night in PAB medium at 30ºC. Stationary phase cultures were processed for microscopy analysis as described in the experimental procedures section. In addition, cultures of wild-type and *ltaS$_{BS}$* mutant *B. subtilis* strains were back diluted 1:100 into fresh PAB medium and grown for 3 hours at 37ºC (exponential phase) and subsequently processed for microscopy. Images were taken with a Zeiss Axiovert 200 wild-field microscope using a 100x objective and analyzed using the Improvision Volocity software.
